# Supplementary material for: Mechanism of integrin activation by talin and its cooperation with kindlin
Source: Nat Commun. 2022 Apr 29;13:2362. doi: 10.1038/s41467-022-30117-w (PMC9054839; doi:10.1038/s41467-022-30117-w)
Supplement: Supplementary file 1 — Supplementary information [file 41467_2022_30117_MOESM1_ESM.pdf]

Supplementary Materials

Mechanism of integrin activation by talin and its cooperation with kindlin

Fan Lu[1,2#], Liang Zhu[1#], Thomas Bromberger[3#], Jun Yang[1], Qiannan Yang[1], Jianmin Liu[1], Edward F. Plow[1], Markus Moser[3]*, and Jun Qin[1,2] *

Fourteen supplementary figures and three supplementary tables

Followed by uncropped images

Supplementary Figure 1 Regulation of integrin activation by talin.

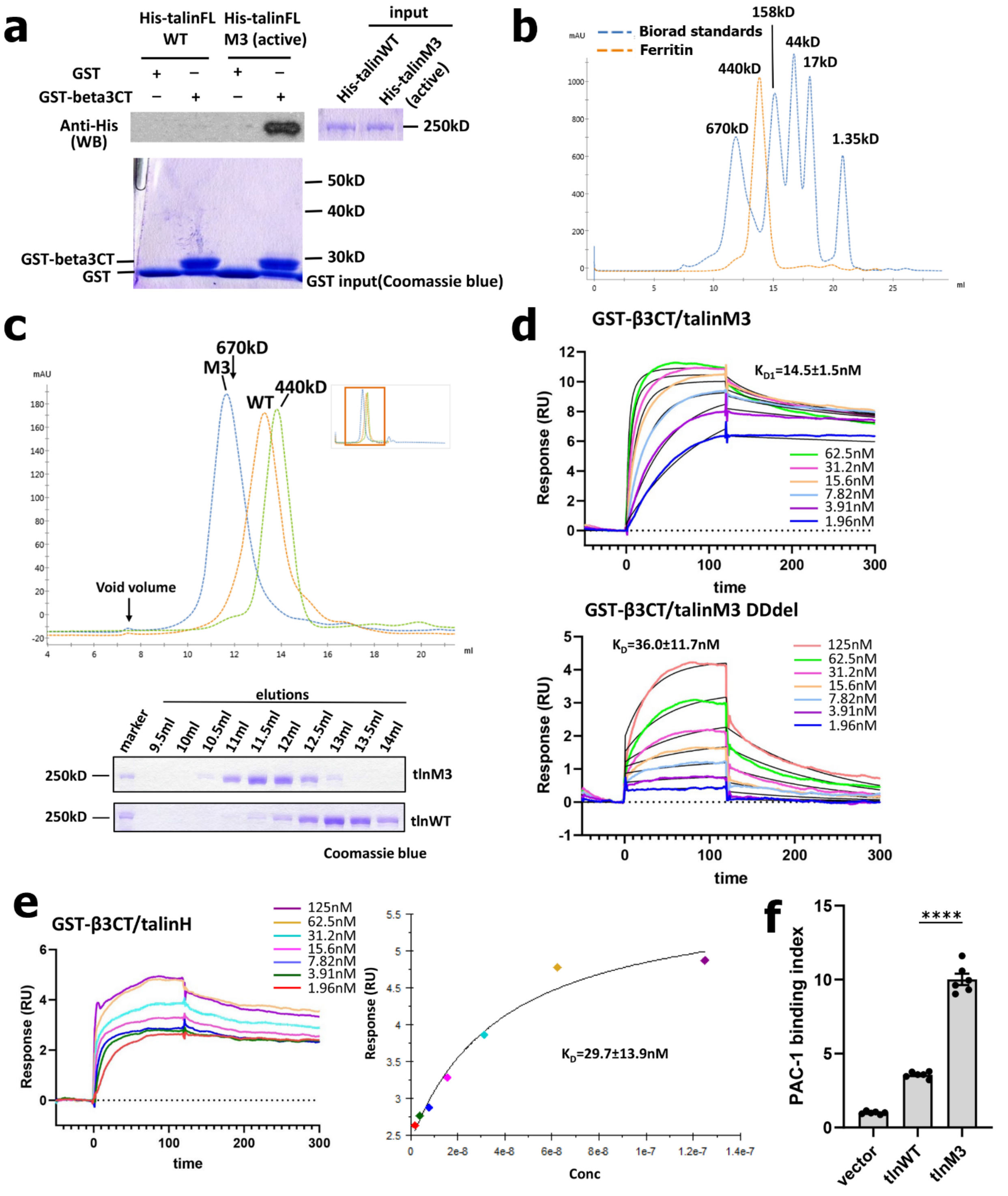

(**a**) Triple mutations (M3) designed on the interface between talinF3 and R9 release talin from auto-inhibited state. As shown in the blot, full-length active talin (talinFL M3) binds to integrin cytoplasmic tail (GST-beta3CT) potently, while full-length wild type talin (talinFL WT) shows little binding to integrin. Two independent experiments were performed. (**b**) Gel filtration of protein markers on Superose

6 10/300 GL column. (**c**) Gel filtration of purified His-tlnM3 FL and His-talinWT FL overlaid with 440 kDa marker, indicating talinWT may form some dimer under 150mM salt while tlnM3 is released from autoinhibited compact conformation with active mutations. Bottom panel: SDS-PAGE of elution fractions from gel filtration show that both tlnM3 and talinWT are pure full length talin, but the conformation and dimerization state make them elute very differently. Two independent experiments were performed. (**d**) SPR sensorgram of tlnM3 FL (top panel) and talinM3 DDdel (bottom panel) when flowing over the surface with immobilized GST-integrinβ3CT. Compared to tlnM3, tlnM3 DDdel lacks C-terminal dimerization domain (DD) which was reported to disrupt talin dimerization efficiently[1]. As a result, full length tlnM3 binding to integrin was best described by a bivalent binding model whereas tlnM3 DDdel (monomeric) affinity to integrin fell well into 1:1 fitting. TlnM3 DDdel affinity to integrin was almost comparable to talinH, whereas tlnM3 binds stronger to integrin than talin-H and tlnM3 DDdel probably due to the bivalent binding of tlnM3 dimer to two immobilized integrins (note GST is also known to form dimer). (**e**) SPR sensorgram of talinH when flowing over the surface with immobilized GST-integrinβ3CT. The affinity was calculated by steady state model. (**f**) tlnM3 induced much higher PAC-1 binding in CHO cells than talinWT indicating the constitutively active state caused by triple mutations. ****, $p<0.0001$ with 95% confidence interval 5.574 to 7.339 (t test), N=6 biologically independent samples. Values are given as mean ± S.E.M. Uncropped images(**a**, **c**) are provided at the end of this file. Raw data (**e**-**f**) are provided in Source Data file.

Supplementary Figure 2 Talin dimerization contributes to enhanced integrin activation and cell adhesion.

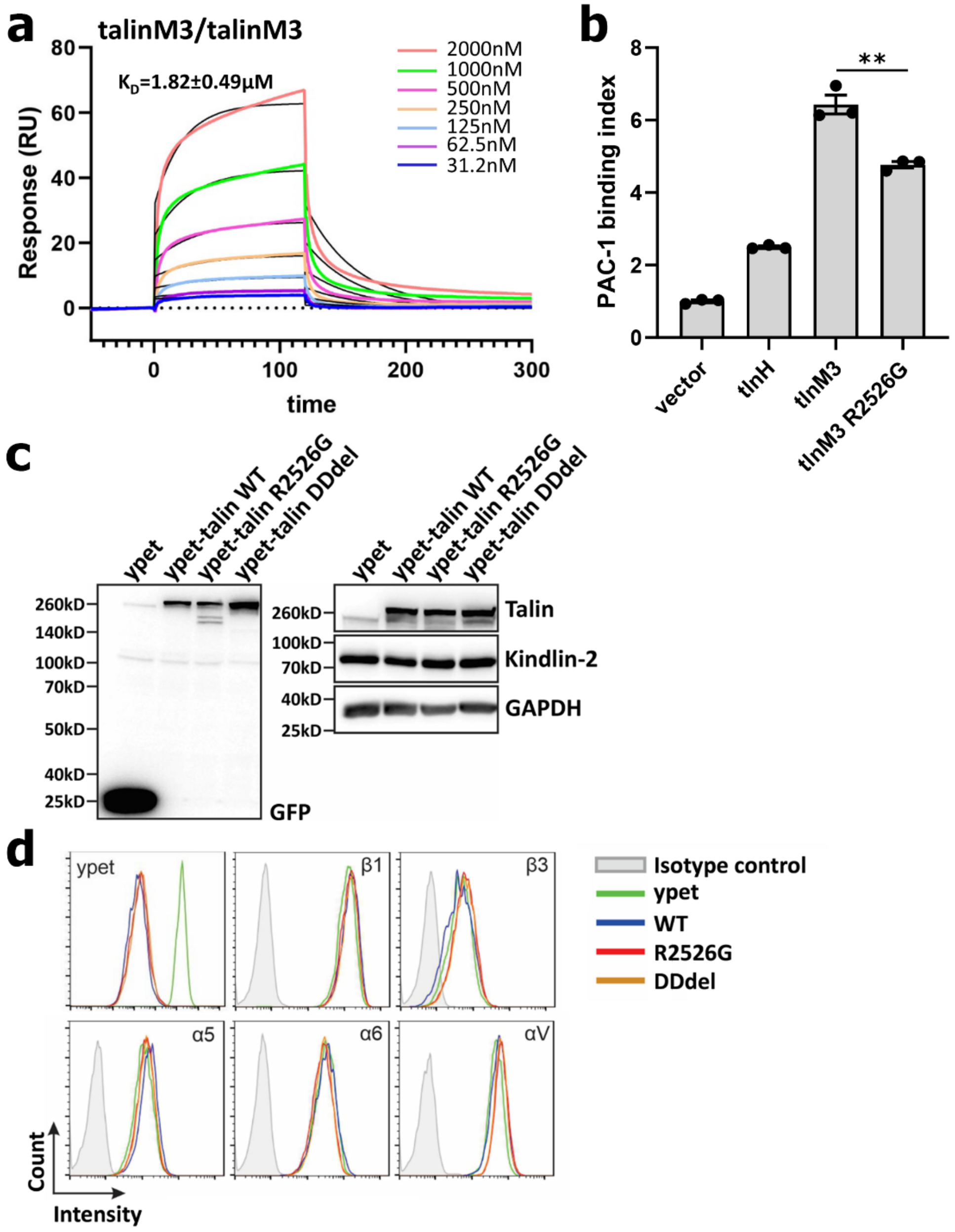

(**a**) SPR sensorgram of full length tlnM3 when flowing over the surface with immobilized tlnM3. Affinity was measured by 1:1 fitting. (**b**) PAC-1 binding assay showing that point mutation R2526G to disrupt talin dimerisation significantly reduces the integrin activation. **, p=0.0037 with 95% confidence interval -2.417 to -0.901 (t test), N=3 biologically independent samples. Values are given as mean ± S.E.M. (**c**) Western blot showing similar amounts of talin and talin mutants were introduced in talin1/2dKO fibroblasts, and the mutations also did not alter kindlin-2 expression level. GAPDH served as loading control. Expression levels were analyzed in 1 experiment. (**d**) FACS analysis of ypet levels to confirm similar cellular levels of talin WT and mutants, and cell surface expression of integrins were not affected

much by talin WT or mutant expression. Uncropped images(**c**) are provided at the end of this file. Raw data(**b**) are provided in Source Data file.

Supplementary Figure 3 Paxillin bridges talin with kindlin-2.

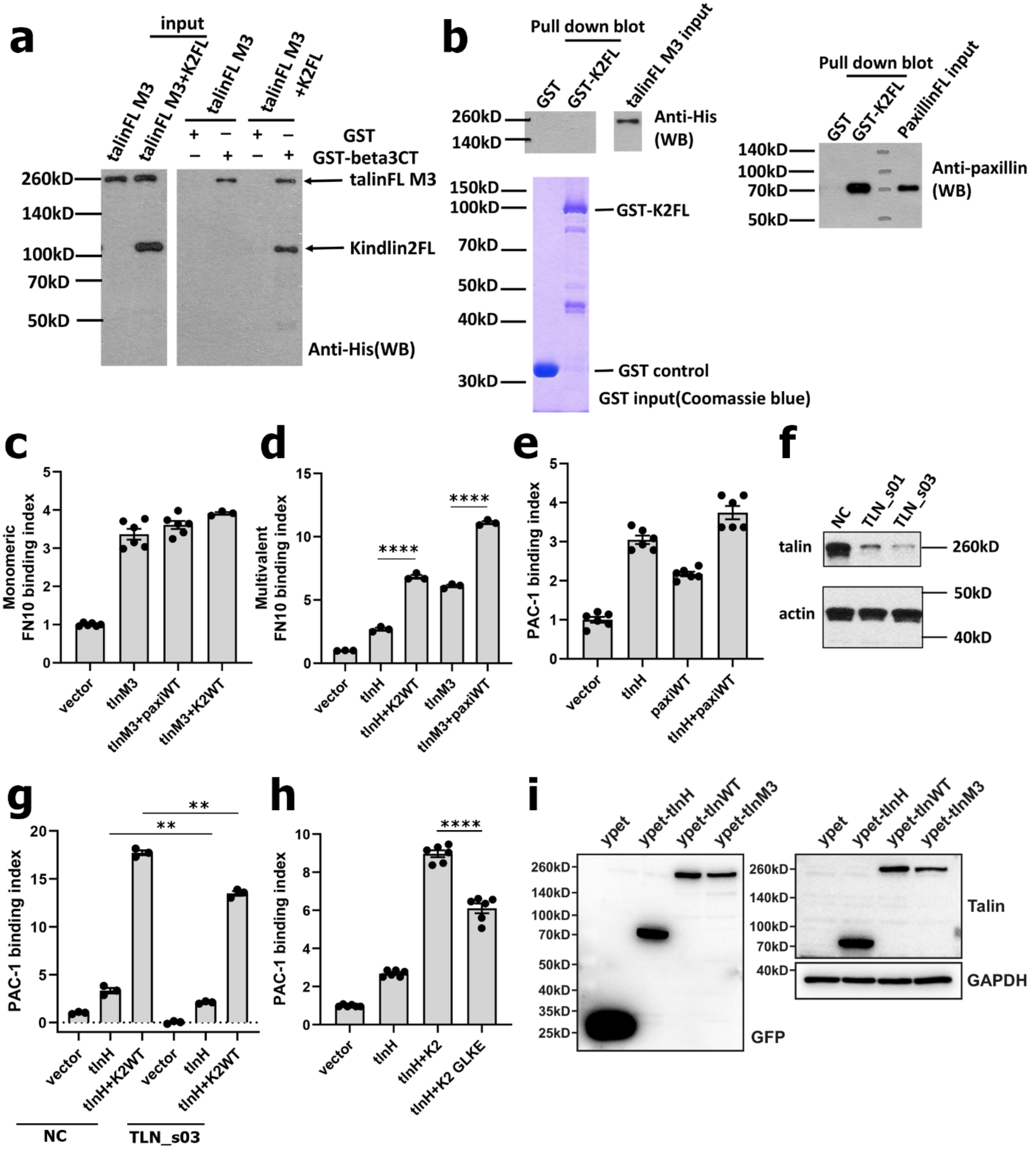

(**a**) Full length active talin (talinFL M3) was pulled down by GST tagged integrin β3 cytoplasmic tail (beta3CT) similarly with and without the same amount of kindlin-2, indicating that talin and kindlin can bind to integrin in a non-exclusive manner. Two independent experiments were performed. (**b**) GST tagged full length kindlin-2 (GST-K2FL) does not pull down full length active talin (tlnM3), indicating that kindlin-2 does not physically interact with talin, whereas GST-K2FL pulls down paxillin efficiently under the same experimental condition. Two independent experiments were performed. (**c**) Paxillin

(paxiWT) does not significantly increase tlnM3-induced monomeric FN10 binding as much as PAC-1 binding. N=6 biologically independent samples. Values are shown as mean ± S.E.M. (**d**) Both tlnH/K2 and tlnM3/paxi co-expression induced synergistic binding of oligomeric His-FN10-biotin that contains the exactly the same binding unit as monomeric FN10, which is consistent with the multivalent PAC-1 binding data in Fig 3A, further supporting the mechanism of microclustering of integrins to bind multivalent ligands. ****, $p<0.0001$, N=3. Values are shown as mean ± S.E.M. (**e**) Paxillin does not cooperate with talin head in integrin activation shown by PAC-1 binding. N=6 biologically independent samples. Values are shown as mean ± S.E.M. (**f**) Western blots showing talin-1 knockdown efficiency. Two independent experiments were performed. (**g**) Knockdown of endogenous talin-1 significantly reduced talinH and kindlin synergy reflected by PAC-1 binding. **, $p=0.0032$ with 95% confidence interval -2.498 to -0.9761 (t test) or 0.0015 with 95% confidence interval -8.444 to -4.002 (t test), N=3 biologically independent samples. Values are shown as mean ± S.E.M. (**h**) Synergy between talinH and kindlin-2 can also be significantly reduced by paxillin binding deficient mutation G42K/L46E. ****, $p<0.0001$ with 95% confidence interval -3.566 to -2.158 (t test), N=6 biologically independent samples. Values are given as mean ± S.E.M. (**i**) Expression levels of ypet, tlnH, tlnWT and tlnM3 in talin1/2dKO fibroblasts assessed by Western blot analysis. GAPDH served as loading control. Expression levels were analyzed in 1 experiment. Uncropped images (**a-b**, **f**, **i**) are provided at the end of this file. Raw data (**c-e**, **g-h**) are provided in Source Data file.

Supplementary Figure 4 Paxillin-kindlin interaction promotes cell adhesion.

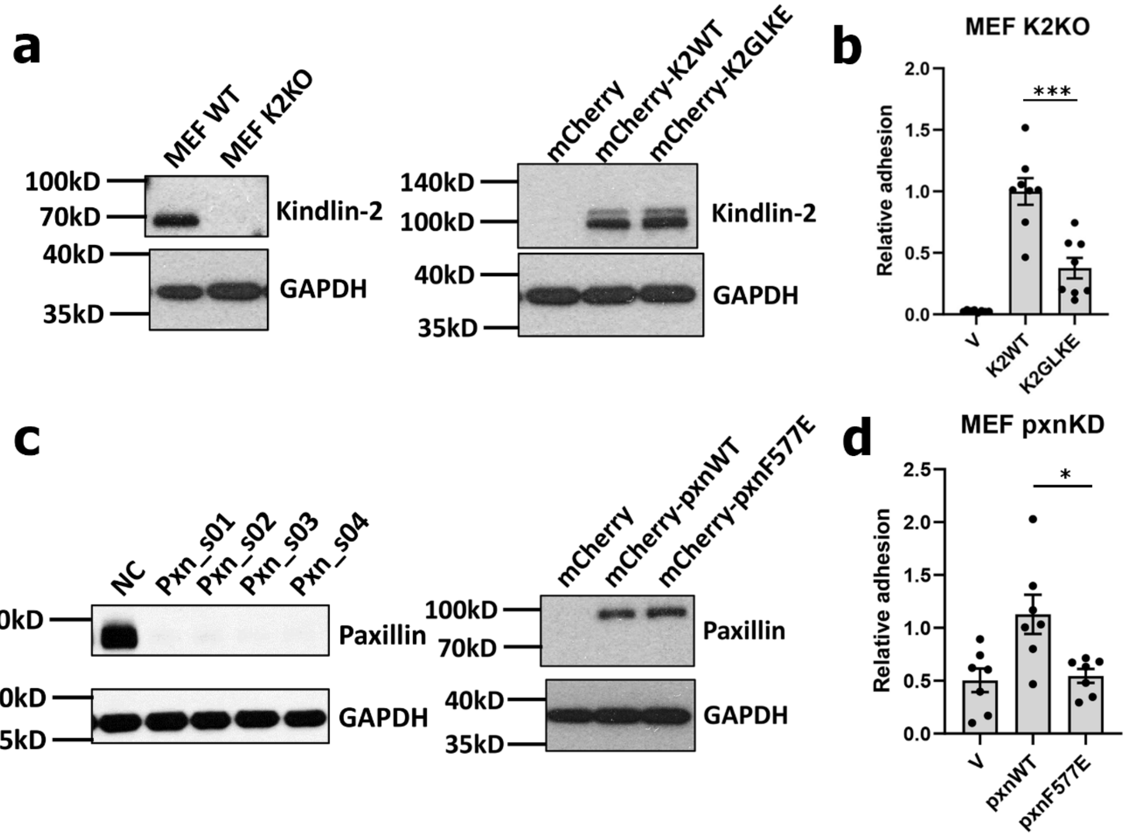

(**a**) Left panel shows the western blot for the complete kindlin-2 knockout in MEF K2KO and right panel shows the western blot for the similar expression levels of mCherry tagged wildtype kindlin-2 (K2WT) and paxillin binding defective mutant (K2GLKE56) in the MEFs K2KO. Two independent experiments were performed. (**b**) Kindlin-2 GLKE mutation significantly reduces the cell adhesion. MEF K2KO cells were transfected with mCherry vector, mCherry tagged K2WT and mCherry tagged K2GLKE. 24 hours post transfection, cells were seeded on to fibronectin coated 96-well plate for 30 min, double washed with DPBS, fixed and stained for quantification. Quantification results are plotted as mean ± SEM. ***, p=0.0004 with 95% confidence interval -0.9165 to -0.3308 (t test), N=8 biologically independent samples. (**c**)Western blot to show efficient paxillin knockdown after siRNA treatment. Pxn_s04 was then selected for knockdown-related experiments. Similar level of mCherry tagged wildtype paxillin(pxnWT) and kindlin binding mutant (pxnF577E) were introduced to the MEF cells after paxillin knockdown. Two independent experiments were performed. (**d**) Paxillin F577E mutation significantly reduces the cell adhesion. MEF cell line from ATCC was treated with paxillin targeting siRNA(pxn_s04) for 48 hours, and followed by mCherry vector, mCherry tagged pxnWT and mCherry tagged pxnF577E transfection. 24 hours post transfection, cells were seeded on to fibronectin coated 96-well plate for 20 min, double washed with DPBS, fixed and stained for quantification. Quantification results are plotted as mean ± SEM. *, p=0.012 with 95% confidence interval -1.012 to -0.1535 (t test), N=7 biologically independent samples. Uncropped images (**a**, **c**) are provided at the end of this file. Raw data (**b**, **d**) are provided in Source Data file.

Supplementary Figure 5 Paxillin 1-160 plays a major role in the talin/paxillin interaction by binding to talin-R.

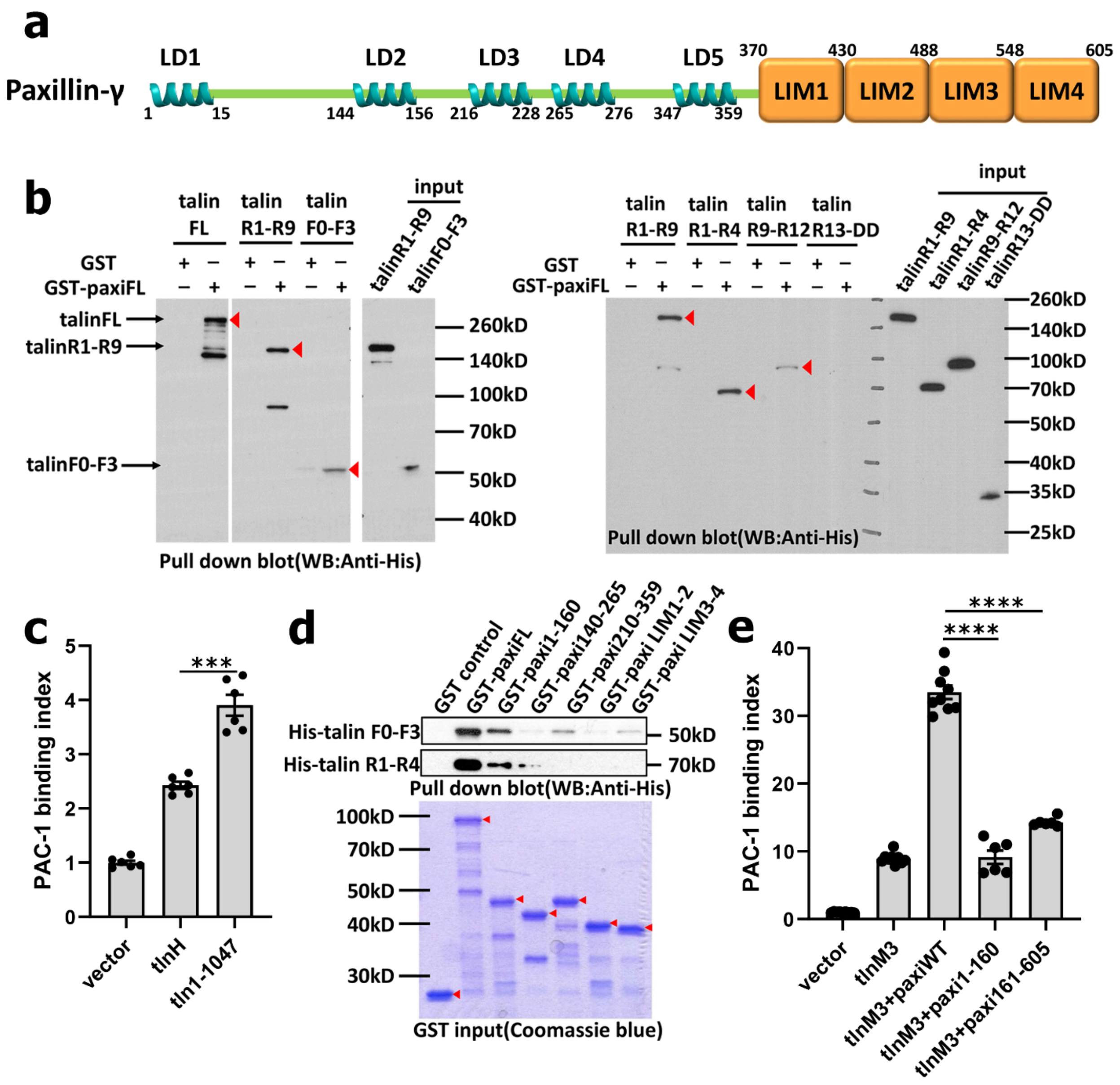

(**a**) Domain organization of paxillin-γ. N-terminal contains five LD motifs connected by flexible linkers, followed by four LIM domains on its C-terminal. (**b**) GST tagged full length paxillin(paxiFL) binds much stronger to talin-R1-R9 than to talin head. Talin-R1-R4 forms the major binding domain for paxillin whereas talin-R9-R12 shows weak binding. Two independent experiments were performed. (**c**) Talin 1-1047 containing talin-H and talin-R1-R4 induces integrin activation more potently than talin-H as shown by PAC-1 binding. ***, p=0.0007 with 95% confidence interval 0.6693 to 1.230 (t test), N=6 biologically independent samples. (**d**) GST-based pull down assays showing that the paxillin binding to talin-R and talin-H are primarily mediated by paxillin 1-160. Two independent experiments were performed. (**e**). Deletion of either paxillin 1-160 or 161-605 drastically reduces the ability of paxillin to synergize with tlnM3 to activate integrin as shown by PAC-1 binding. ****, p<0.0001 with 95% confidence interval -

27.55 to -21.13 (t test) or with 95% confidence interval -21.94 to -16.44 (t test), N=6 biologically independent samples. Values are shown as mean ± S.E.M. Uncropped images (**b**, **d**) are provided at the end of this file. Raw data (**c**, **e**) are provided in Source Data file.

Supplementary Figure 6 Multiple talin rod domain binds to paxillin N-terminal.

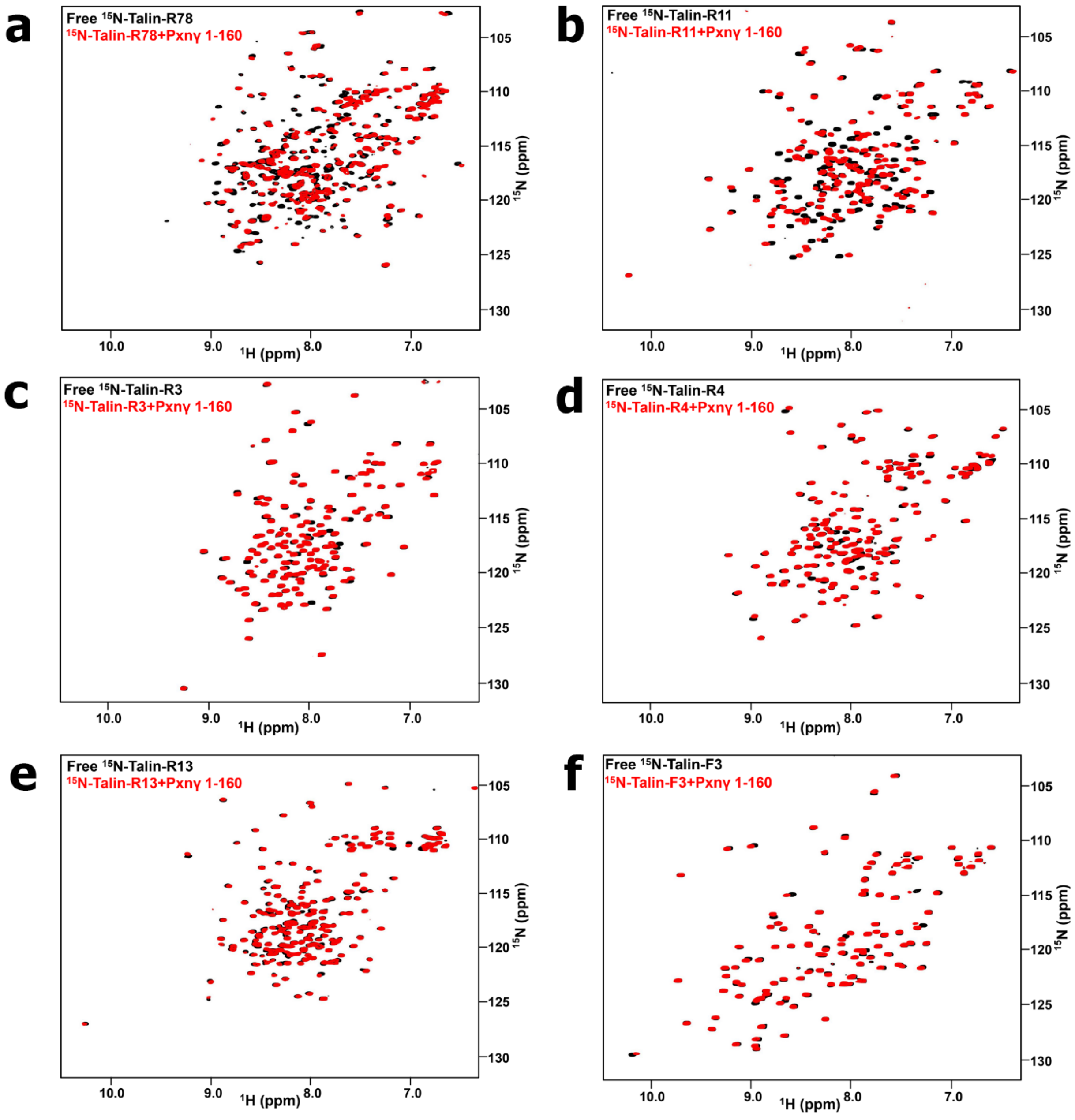

(**a**) HSQC spectra to show Talin-R7-R8 binding to paxillin 1-160; (**b**) HSQC spectra to show Talin-R11 binding to paxillin 1-160. (**c**) HSQC spectra to show Talin-R3 binding to paxillin 1-160; (**d**) HSQC spectra to show Talin-R4 binding to paxillin 1-160. (**e**) HSQC spectra to show Talin-R13 binding to paxillin 1-160; (**f**) HSQC spectra to show Talin-F3 binding to paxillin 1-160. The bindings are all tested with 50µM ^{15}N talin subdomain with 100uM unlabeled paxillin 1-160.

Supplementary Figure 7 TalinR1-R4 is mainly responsible for paxillin binding.

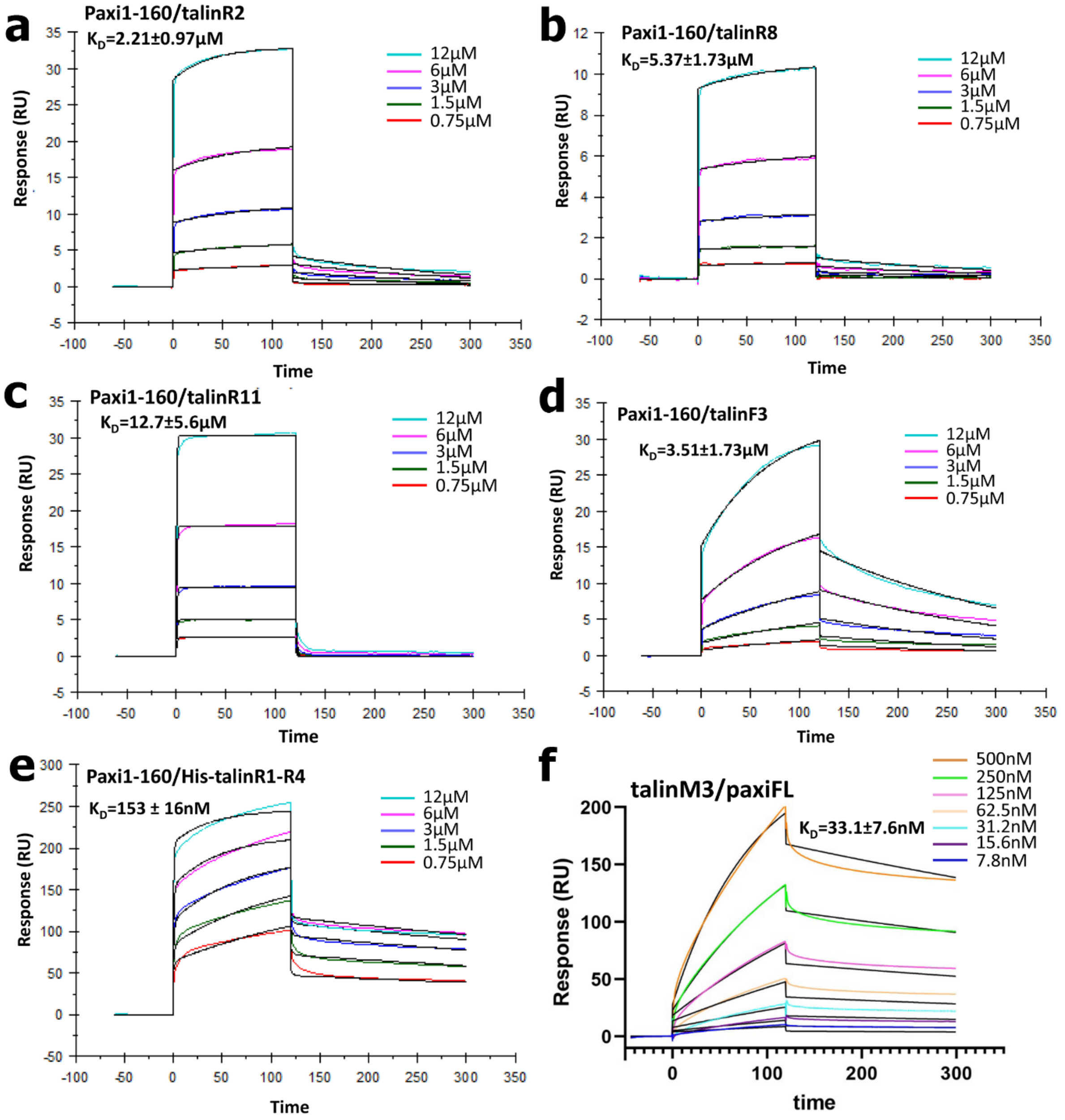

(**a**) SPR sensorgram of talinR2 when flowing over the surface with immobilized paxillin1-160. The affinity was calculated by 1:1 fitting. (**b**) SPR sensorgram of talinR8 when flowing over the surface with immobilized paxillin1-160. The affinity was calculated by 1:1 fitting. (**c**) SPR sensorgram of talinR11 when flowing over the surface with immobilized paxillin1-160. The affinity was calculated by 1:1 fitting. (**d**) SPR sensorgram of talinF3 when flowing over the surface with immobilized paxillin1-160. The affinity was calculated by 1:1 fitting. (**e**) SPR sensorgram of talinR1-R4 when flowing over the surface with immobilized paxillin1-160. The binding event was best described by two state model (still 1:1 binding), indicating some conformational change of talin-R1-R4 may occur to engage the cooperative binding of talin-R2, R3, and R4 to paxillin 1-160. Conformational change of talinR1-R4 may explain the greatly enhanced binding in comparison to talinR2 alone. (**f**) SPR sensorgram of full length paxillin when

flowing over the surface with immobilized full length tlnM3. Paxillin samples were kept at 10ºC before injection to prevent degradation. The affinity was calculated by 1:1 fitting, indicating talin R1-R4 mainly contributes to the talin-paxillin interaction. All tests were performed at 25ºC.

Supplementary Figure 8 Paxillin competes with talin-R9 and integrin for binding to talin-H.

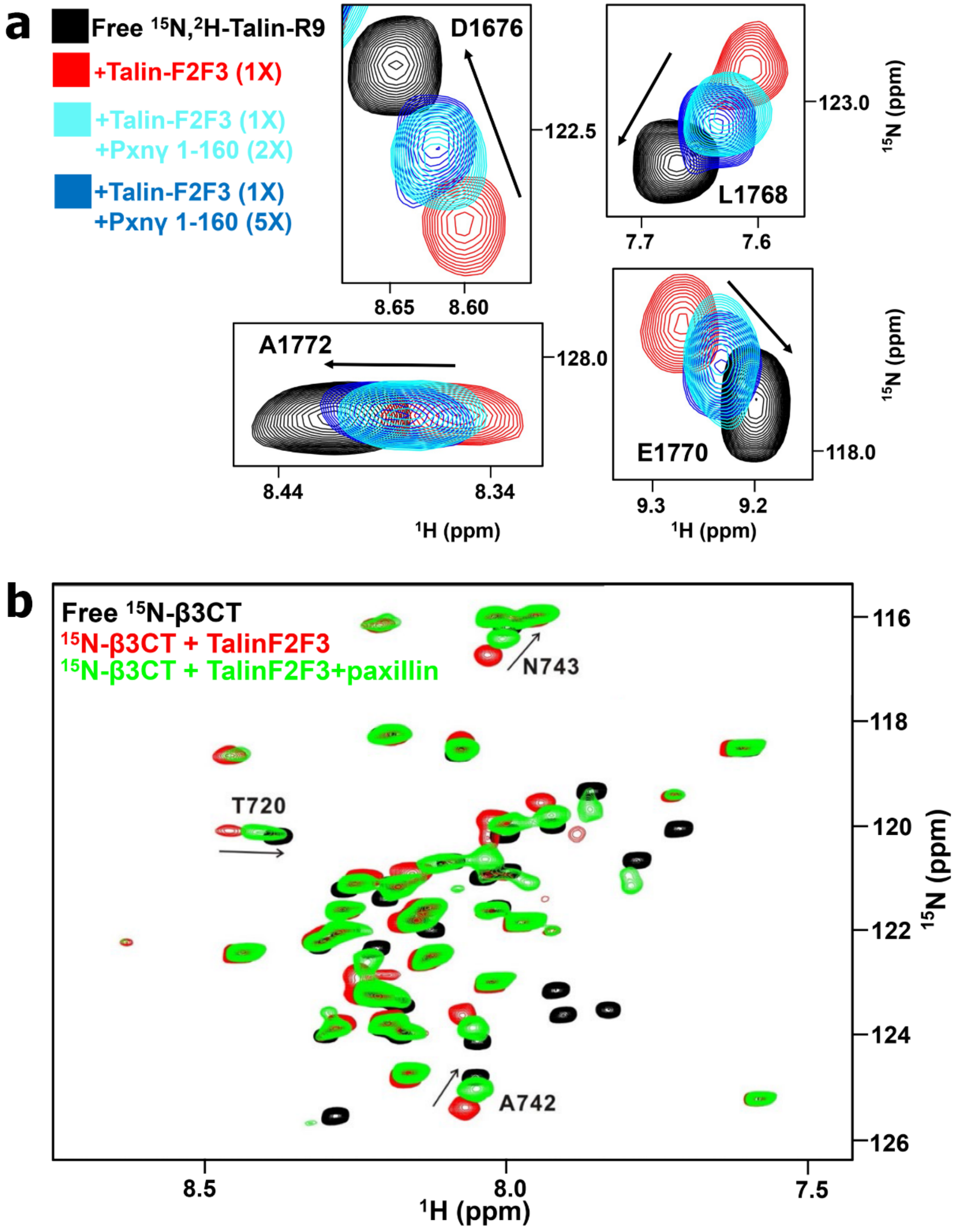

(**a**) Paxillin competes with autoinhibitory talin-R9 for binding to talin-F2F3 of talin-H. 2D-HSQC spectra (selected residues were shown) of 50 μM ^{15}N-^{2}H (80%) labeled Talin R9 in the absence (black) and presence of 50 μM unlabeled talin-F2F3 (red). Addition of 100 μM unlabeled paxillin 1-160 (cyan) or 250 μM paxillin 1-160 (blue) shift the peaks towards the free form talin-R9, showing that paxillin competes with the autoinhibitory talin-R9 for binding to talin-F3. This provides a basis for understanding why paxillin preferably binds to activated talin in Fig 4A. (**b**) Paxillin competes with talinF2F3 binding to integrin β3 CT. 2D HSQC spectra of 50μM ^{15}N labeled integrin β3 CT in absence (black) and presence of

100 μM unlabeled talinF2F3 (red). Addition of 200 μM paxillin 1-160 (green) shift the peaks towards to free form integrin β3 CT, indicating paxillin1-160 and integrin share overlapping binding sites on talin. It should be emphasized that membrane-embedded integrin binds very tightly to membrane-anchored talin in real cellular environment[2], which should readily displace the weak binding of paxillin to talin-F3 (see Supplementary Figure 7d) while strong binding of talin-R/paxillin retain to link with kindlin.

Supplementary Figure 9 Paxillin LD motifs are responsible for binding to talin-R subdomains.

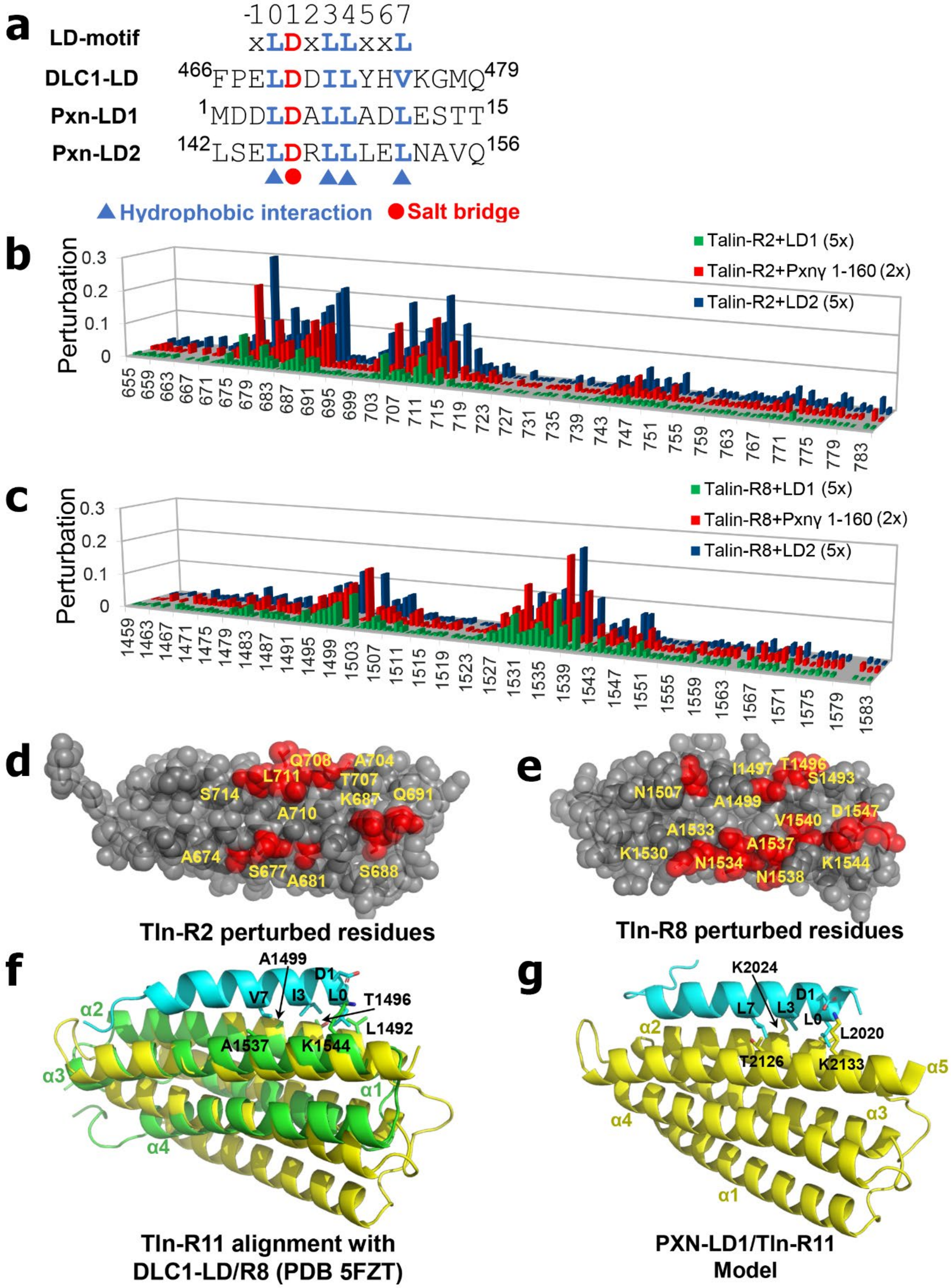

(**a**) Sequence alignment of DLC1-LD and paxillin-LD1/2 motifs. Residues that contribute to the binding of talin-R8 subdomain are highly conserved. (**b**) Chemical shift perturbation of 50 µM talin-R2 by 250 µM paxillin LD1 (green bar) or 100 µM paxillin 1-160 (red bar) or 250 µM paxillin LD2 (blue bar). Note that at the same concentration (250 µM), LD2 clearly binds much stronger to talin-R2 than LD1. We used less amount of paxillin1-160 (100 µM) that contain LD1 and LD2. As expected, the fragment binds less to talin-R2 at talin-R2: paxillin1-160 = 1:2 than LD2 at 250 µM (talin-R2:LD2 = 1:5) but more than LD1 at 250 µM (talin-R2:LD1 = 1:5). (**c**) Chemical shift perturbation of 50 µM talin-R8 by 250 µM paxillin LD1 (green bar) or 100 µM paxillin 1-160 (red bar) or 250 µM paxillin LD2 (blue bar). (**d**) Sphere representation of talin-R2 (extracted from PDB code 1SJ8) and (**e**) talin-R8 (extracted from PDB code 5FZT). The surface-exposed residues with significant chemical shift perturbations were colored in red. The similar pattern in both domains suggests that they may interact with paxillin in the same way. (**f**) Structure alignment of talin-R11 (yellow) with published complex structure of DLC1-LD (cyan) and talin-R8 (green) (PDB code 5FZT). The sidechains of important interfacial residues in the complex are shown and labeled. (**g**) A predicted model of paxillin-LD1 bound to talin-R11 by structurally-aligning talin-R11 (yellow) and paxilline-LD1 (cyan) to the DLC1-LD/talin-R8 complex. The model shows a similar interacting pattern of residues with their sidechains labeled and displayed.

Supplementary Figure 10 Paxillin 1-160 likely uses both LD1 and LD2 motifs to interact with talin R1-R4 via a multi-site binding mode.

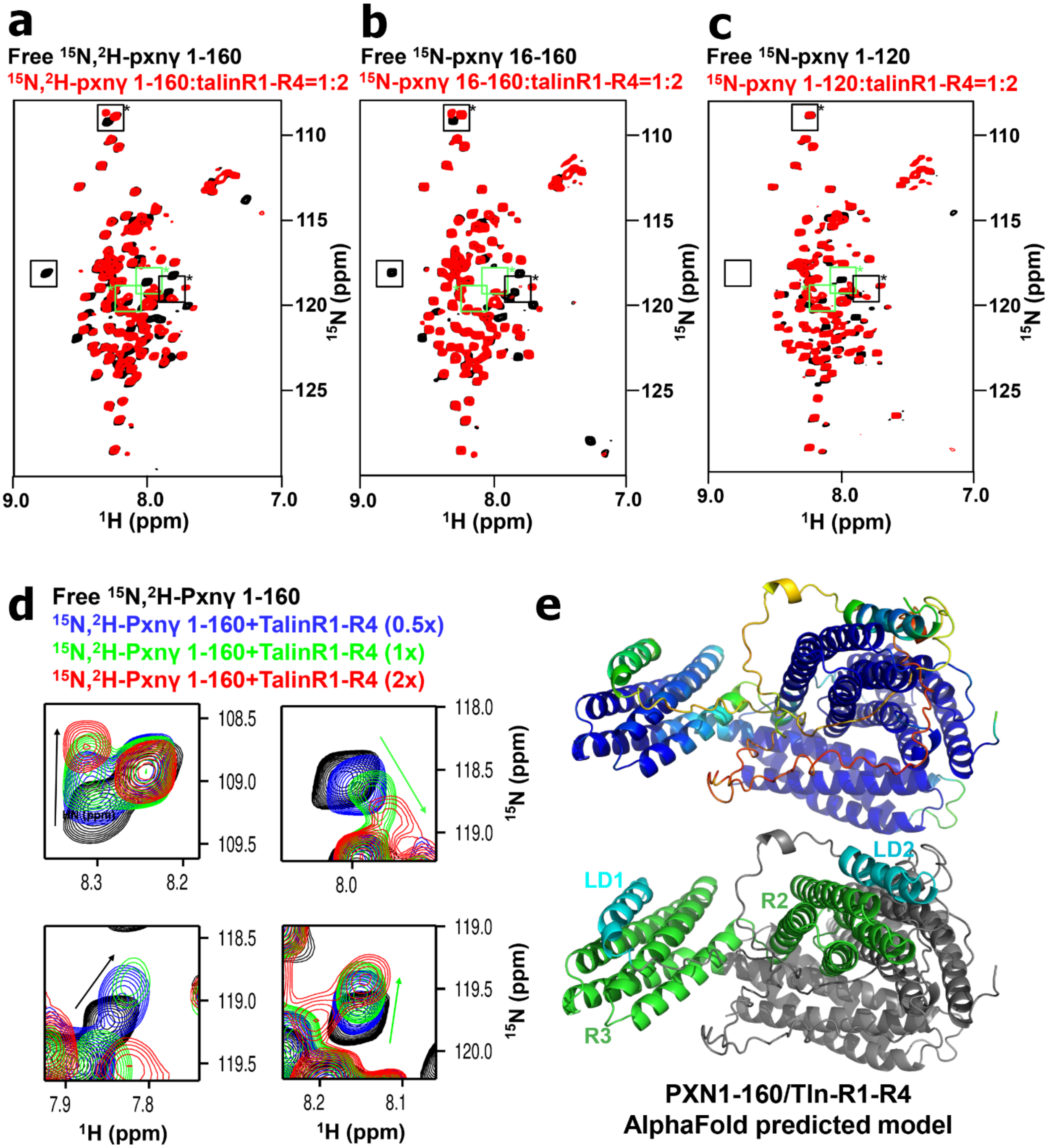

(**a**-**c**) NMR spectra of 50 µM ^{15}N(100%)/^{2}H(80%)-labeled paxillin 1-160 (**a**), paxillin 16-160 (LD1 deletion, **b**), or paxillin 1-120 (LD2 deletion, **c**) with 100 µM unlabeled talin R1-R4. The residues enclosed with black boxes are judged from LD2 motif since they were visible in both panel **a** and **b** but missing in panel **c**, and the residues enclosed with green boxes are likely from LD1 motif since they were visible in both panel **a** and **c** but missing in panel **b**. (**d**) Individually-zoomed regions in panel **a** of the

boxes with an asterisk. The two left panels are residues from LD2 motif (shown as black boxes) and two right panels are residues from LD1 motif (shown as green boxes). The arrow indicates the trends with increasing amount of talin R1-R4 addition. (**e**) Cartoon representation of the best complex structure of talin R1-R4 and paxillin 1-160 through Google's AlphaFold2 prediction. The color scheme of the top panel shows the ascending confidence level of the prediction with the low-to-high order as red-yellow-green-cyan-blue. The bottom panel shows the identical structure with the highlighted regions for paxillin LD motif (cyan) and talin-R2 / R3 (green), indicating a multi-site binding mode.

Supplementary Figure 11 Point mutations on the calculated or predicted binding interface successfully disrupted talin-paxillin interaction.

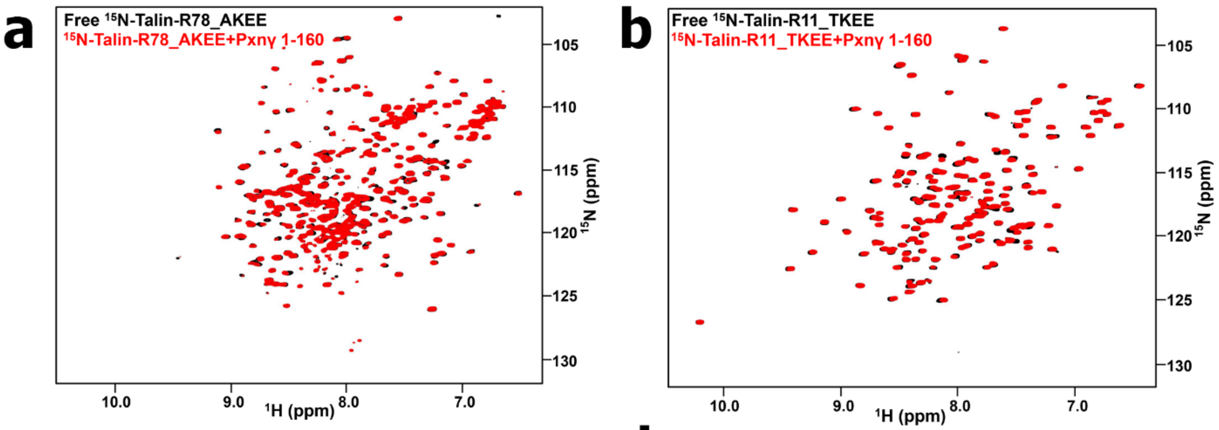

c

Talin-R78	PXN1-160
WT	+
K1544E	reduced
A1537E/K1544E (AKEE)	-

d

Talin-R11	PXN1-160
WT	+
K2133E	reduced
T2126E, K2133E (TKEE)	-

(**a**) Mutations in talin-R8 A1537E/K1544E, AKEE, nearly abolished talin-R7-R8 binding to paxillin 1-160; (**b**) Mutations in talin-R11 T2126E/K2133E, TKEE, nearly abolished the talin-R11 binding to paxillin 1-160. (**c**) Summary of HSQC-based binding of talin R78 and its mutants to paxillin 1-160; (**d**) Summary of HSQC-based binding of talin-R11 and its mutants to paxillin 1-160. All HSQC were performed with 50µM of ^{15}N labeled talin domains with 100µM paxillin 1-160. Binding strengths were estimated by extents of peak shifting.

Supplementary Figure 12 Paxillin functions as an important bridge between talin and kindlin.

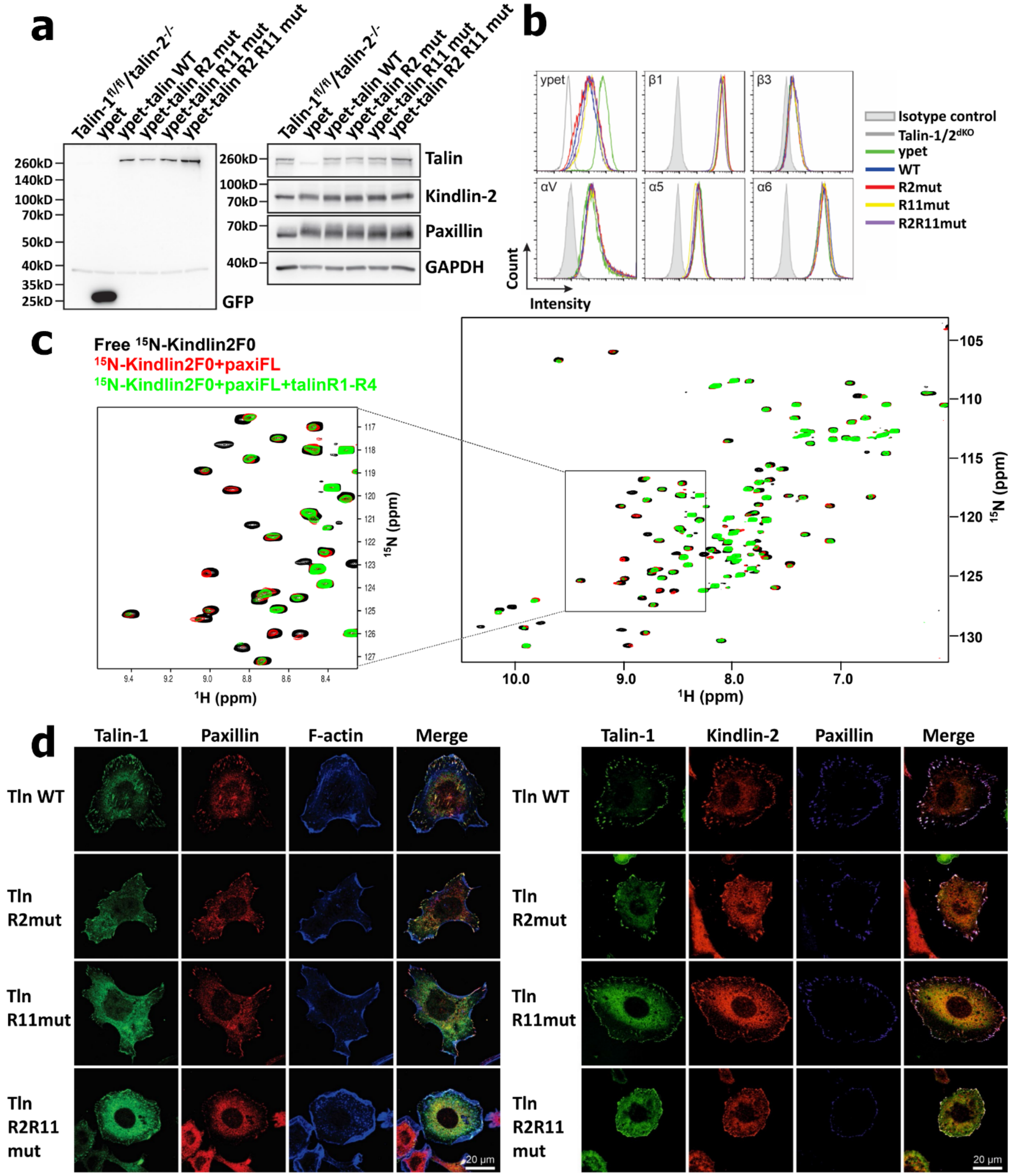

(**a**) Western blot showing similar talin, kindlin-2 and paxillin expression levels in talin1/2dKO cells rescued with WT and paxillin binding-defective talin variants. GAPDH served as loading control. Shown blots are representative of 4 independent experiments. (**b**) Surface expression levels of integrins β1, β3, α5, α6 and αV are comparable on cells expressing ypet-talin WT and mutants as shown by FACS analysis. (**c**) NMR titration of 50 μM ^{15}N-labeled kindlin-2 F0 in the absence (black) or presence of 50

µM full length paxillin (red) or presence of 50 µM full length paxillin and talin R1-R4 (green). The experiments were specifically conducted at 5°C to keep the sample homogenous, but all spectra were obtained at the same condition. Protein quality and quantity were analyzed by SDS-PAGE after each test. Comparison of the red and green spectra shows that additional line-broadening occurs when talin R1-R4 was added to bind paxillin as a result of increasing complex size, suggesting a ternary complex is formed among kindlin2-F0, paxillin, and talin R1-R4. Note that kindlin-2 F0 is only specifically bound to paxillin C-terminal LIM4 as previously determined[3] whereas paxillin N-terminus especially 1-160 is mainly involved in binding to talin-R1-R4 as determined from this study, which forms the basis of this ternary complex formation. (**d**) Confocal images of talin1/2dKO cells expressing ypet-talin WT, talin R2mut (AKEE), talin R11mut (TKEE) and talin R2R11mut (AKEE/TKEE) stained either for paxillin (red) and F-actin (blue; left panel) or kindlin-2 (red) and paxillin (blue; right panel) Scale bar, 20µm. Representative images from 6 (left) and 4 (right) independent experiments are shown (8-10 cells were imaged per experiment). Quantitative characterization of focal adhesions and talin and kindlin-2 localization are shown in Figures 6f+g. Uncropped images(**a**) are provided at the end of this file.

Supplementary Figure 13 Talin-kindlin cooperation is mainly supported by talin dimerization and talin-paxillin-kindlin pathway.

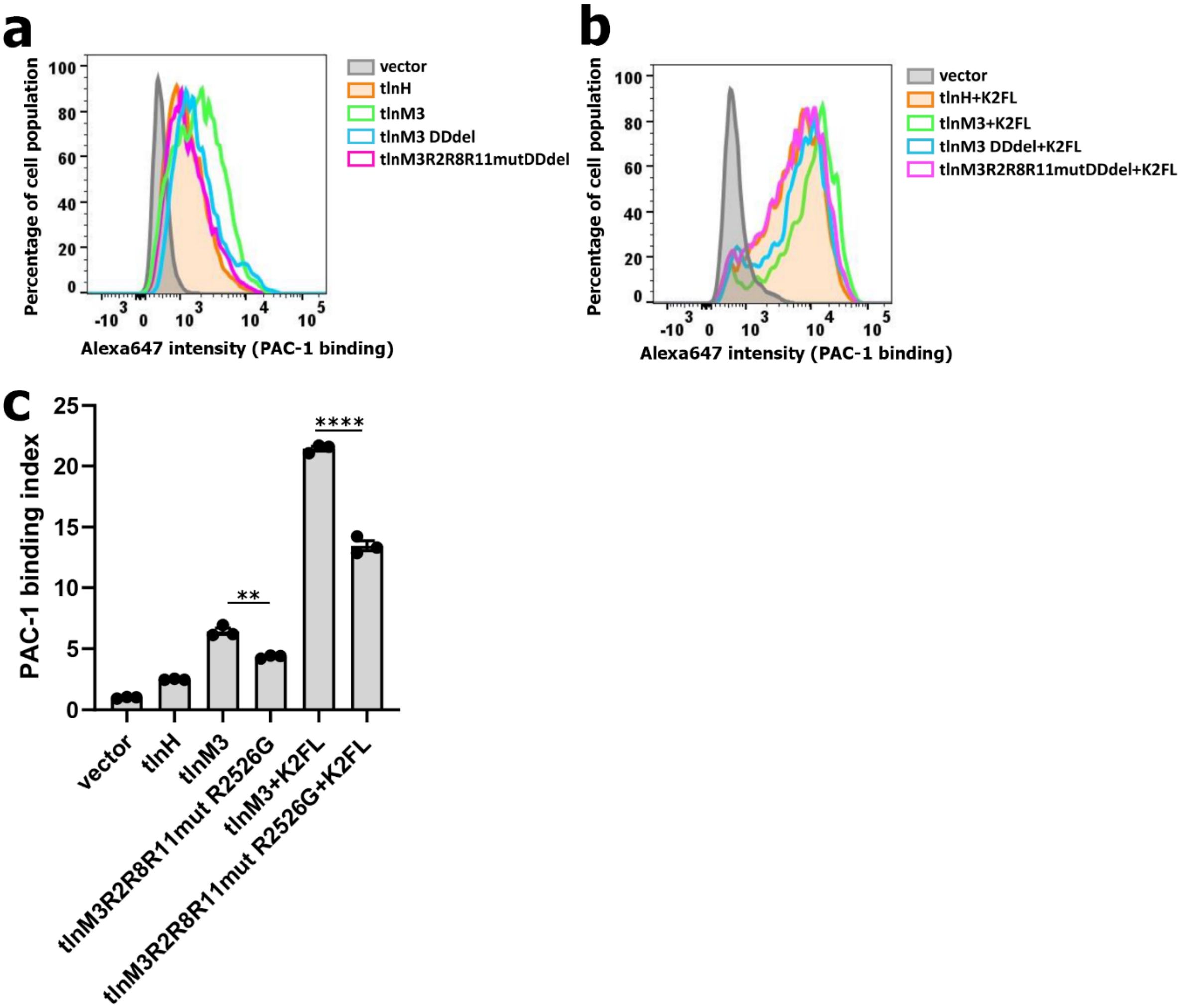

(**a**) Histogram plot of PAC-1 binding to show that deletion of talin dimerization domain (DDdel) reduces full length active talin (tlnM3) induced integrin activation. Addition of paxillin binding defective mutations (R2R8R11mutDDdel) further reduces PAC-1 binding to the level as talin head(tlnH). (**b**) Histogram plot of PAC-1 binding to show that deletion of talin dimerization domain (DDdel) reduces the cooperation between full length active talin and kindlin-2. Addition of paxillin binding defective mutations (R2R8R11mutDDdel) almost abolishes the cooperation between full length talin and kindlin. (**c**) The synergy between tlnM3 and kindlin-2 to activate integrin is substantially reduced by the dimerization mutation of talin R2526G. Values are given as mean ± S.E.M. ** p=0.0015 with 95% confidence interval -2.835 to -1.330 (t test), **** p<0.0001 with 95% confidence interval -9.186 to -6.715 (t test), N=3 biologically independent samples. Raw data (**c**) are provided in Source Data file.

Supplementary Figure 14 Diagram of flow cytometry gating method for CHO cell based integrin activation assays (PAC-1 binding and FN10 binding).

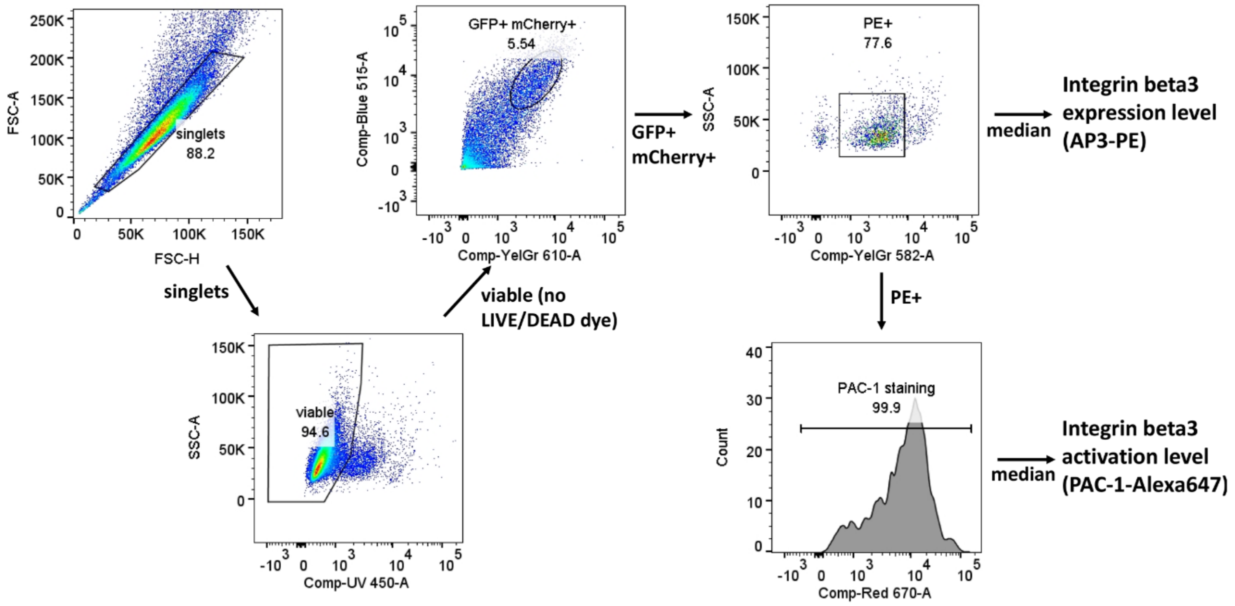

Supplementary Table 1 A summary of binding analysis of different ^{15}N -labeled talin fragments to paxillin (1-160) by 2D NMR HSQC [a]

Talin	PXN1-160
**F0F1**	**-**
**F1F2**	**-**
**F2F3**	+
**F3**	+
**R1**	**-**
**R2**	++
**R3**	**weak**
**R4**	**weak**
**R5**	**-**
**R6**	**-**
**R7**	**-**
**R8**	++
**R9**	**-**
**R10**	**-**
**R11**	++
**R12**	**-**
**R13**	**weak**

[a] "++", "+" and "weak" were used to indicate the relative binding potency based on the overall chemical shift changes of ^{15}N-labeled talin fragments ("++" > "+" > "weak"). "-" indicates no obvious chemical shift change was observed.

Supplementary Table 2 A summary of binding analysis of wild-type and mutant ^{15}N-labeled talin fragments (R2, R8 and R11) to paxillin 1-160 or LD motifs (LD1 and LD2) by 2D NMR HSQC [a]

Talin	PXN1-160	LD1	LD2
**R2**	++	+	+
**R8**	++	+	+
**R11**	++	+	+
**R2 AKEE**	**-**	**NA**	**NA**
**R8 AKEE**	**-**	**NA**	**NA**
**R11 TKEE**	**-**	**NA**	**NA**

[a] "++" and "+" were used to indicate the relative binding potency based on the overall chemical shift changes of ^{15}N-labeled talin fragments ("++" > "+"). "-" indicates no obvious chemical shift change was observed.

Supplementary Table 3. HADDOCK statistics of clustering

Cluster	Size	Haddock Score	Energy (kcal/mol) VDW	Electrostatic	Desolvation	Restrain Viol.	Buried Surface Area (Å^2)
1	38	-69±3	-45±3	-125±8	-6.6±3.4	78±26	1478±30
2	34	-65±3	-45±2	-113±10	-5.1±2.1	76±36	1495±60
7	7	-62±3	-43±7	-88±34	-6.9±2.4	58±17	1484±56
5	11	-52±5	-30±3	-127±9	-4.4±1.7	75±26	1243±75
3	16	-49±4	-32±9	-78±24	-7.3±1.3	63±19	1289±135
9	6	-44±3	-22±2	-125±8	-1.3±1	42±25	1069±54
6	7	-43±8	-26±8	-98±55	-7.4±3.1	94±31	1123±122
4	12	-39±2	-24±3	-100±5	-3.1±1.5	84±52	1036±52
11	5	-39±6	-31±5	-48±20	-5.2±2	77±33	1186±72
10	5	-38±3	-32±2	-57±8	-2.6±2	79±30	1144±48
8	6	-29±5	-26±5	-33±8	-5.8±1.6	88±47	1093±117

Supplementary Table 4. Primer sequence information

Plasmid construct	Primer sequence (5' - 3')
pHis1-talinR1 (483-659)	Forward (EcoRI): ATT CCG GAA TTC GGA CAC ATG CCA CCT CTG<br>Reverse (XhoI): TAT CCG CTC GAG TTA ACT TTC CCC AAT TTG CTG
pHis1-talinR2 (653-789)	Forward (EcoRI): ATT CCG GAA TTC CAG CAA ATT GGG GAA AGT G<br>Reverse (XhoI): TAT CCG CTC GAG TTA GGC GTG GGC CTT CAC GTG
pHis1-talinR3 (783-913)	Forward (EcoRI): ATT CCG GAA TTC CAG CAC GTG AAG GCC CAC<br>Reverse (XhoI): TAT CCG CTC GAG TTA CAA CTT CTT CTT GAT GGC
pHis1-talinR4 (907-1047)	Forward (EcoRI): ATT CCG GAA TTC AAC GCC ATC AAG AAG AAG<br>Reverse (XhoI): TAT CCG CTC GAG TTA AGG TCC ACA TGC CTC CTG
pHis1-talinR5 (1040-1207)	Forward (EcoRI): ATT CCG GAA TTC AAG GCT CAG GAG GCA TGT GGA<br>Reverse (SalI): ATA CGC GTC GAC TTA TCT TTG GCC AGG CAG GCA GCT
pHis1-talinR6 (1202-1357)	Forward (EcoRI): ATT CCG GAA TTC TGC CTG CCT GGC CAA AGA GAC<br>Reverse (XhoI): TAT CCG CTC GAG TTA TGC CTG CTG GGT GCA CAT GGT
pHis1-talinR7-8 (1357-1655)	Forward (EcoRI): ATT CCG GAA TTC GCA CCT GGC CAG AAG GAG TGT<br>Reverse (XhoI): TAT CCG CTC GAG TTA GGC TTT GTC CCT CAT GCT TGT
pHis1-talinR8 (1458-1586)	Forward (EcoRI): ATT CCG GAA TTC CAG CAA GGA CTG GTG GAA CCC<br>Reverse (XhoI): TAT CCG CTC GAG TTA GGC AGG GAC GCT GGA GAA CT
pHis1-talinR10 (1818-1975)	Forward (EcoRI): ATT CCG GAA TTC GCC AGT GCT GCA GGA GTC GTT<br>Reverse (XhoI): TAT CCG CTC GAG TTA GGT ACC ACG ATT CCC AGC CTG
pGST1-paxillin 16-160	Forward (EcoRI): ATT CCG GAA TTC TCC CAC ATC TCC AAA CGG<br>Reverse (XhoI): TAT CCG CTC GAG TTA TGG CGG GTT ATG CTG TAC AGC
pGST1-paxillin 1-120	Forward (EcoRI): ATT CCG GAA TTC ATG GAC GAC CTC GAC GCC CT<br>Reverse (XhoI): TAT CCG CTC GAG TTA GAA GCT GTA GAC GTG CTC
pGST1-paxillin 161-605	Forward: ATG GAT CCG GAA TTC GGC TTC CCT GCA GAT<br>Reverse: ATC TGC AGG GAA GCC GAA TTC CGG ATC CAT
pGST1-FN10 (1541-1639)	Forward (EcoRI): ATT CCG GAA TTC GTT TCT GAT GTT CCG AGG<br>Reverse (XhoI): TAT CCG CTC GAG TTA CAT CTG GGA TGG TTT GTC
pmCherry-c1 paxillin1-605	Forward (XhoI): TAT CCG CTC GAG CT ATG GAC GAC CTC GAC GCC CTG<br>Reverse (EcoRI): ATT CCG GAA TTC TTA GCA GAA GAG CTT GAG GAA
pmCherry-c1 paxillin1-160	Forward: GTA CAG CAT AAC CCG CCA TAA GGC TTC CCT GCA GAT GAG<br>Reverse: CTC ATC TGC AGG GAA GCC TTA TGG CGG GTT ATG CTG TAC

pmCherry-c1 paxillin161-605	Forward: GGA CTC AGA TCT CGA GCT GGC TTC CCT GCA GAT GAG<br>Reverse: CTC ATC TGC AGG GAA GCC AGC TCG AGA TCT GAG TCC
talinM3 (M319A T1767L E1770K)	Forward: CTA GTC AAG GAA AAG GCG AAG GGG AAG AAT AAA C<br>Reverse: GTT TAT TCT TCC CCT TCG CCT TTT CCT TGA CTA G<br>Forward: ACT AAA CTG TTG GCA AAG TCT GCC TTG CAG TTG<br>Reverse: CAA CTG CAA GGC AGA CTT TGC CAA CAG TTT AGT
pEGFP-c1 talin F0-R4 (1-1047)	Forward (XhoI): TAT CCG CTC GAG C ATG GTT GCG CTT TCG CTG<br>Reverse (EcoRI): ATT CCG GAA TTC TTA AGG TCC ACA TGC CTC CTG
talinR2 AKEE (A680E K687E)	Forward: TGG CAA GTG CTG CAG AGG CCC TGG TCC TCA AG<br>Reverse: CTT GAG GAC CAG GGC CTC TGC AGC ACT TGC CA<br>Forward: CTG GTC CTC AAG GCC GAG AGT GTG GCC CAG C<br>Reverse: GCT GGG CCA CAC TCT CGG CCT TGA GGA CCA G
talinR8 AKEE (A1537E K1544E)	Forward: GTG GCC AAC AGT ACA GAG AAT CTT GTC AAG ACC A<br>Reverse: TGG TCT TGA CAA GAT TCT CTG TAC TGT TGG CCA C<br>Forward: CTT GTC AAG ACC ATC GAG GCA CTA GAT GGG G<br>Reverse: CCC CAT CTA GTG CCT CGA TGG TCT TGA CAA G
talinR11 TKEE (T2126E K2133E)	Forward: G ATG GTG ACC AAT GTG GAA TCA TTG CTC AAG ACA<br>Reverse: TGT CTT GAG CAA TGA TTC CAC ATT GGT CAC CAT C<br>Forward: TTG CTC AAG ACA GTG GAG GCT GTG GAA GAT G<br>Reverse: CAT CTT CCA CAG CCT CCA CTG TCT TGA GCA A
talinDDdel	Forward: GCA GCC TTC GAA GAC TAG GAG AAT GAG ACG G<br>Reverse: CCG TCT CAT TCT CCT AGT CTT CGA AGG CTG C
talinR2526G	Forward: AGC TCG CCC AGA TCG GGC AGC AGC AGT AC<br>Reverse: GTA CTG CTG CTG CCC GAT CTG GGC GAG CT
pLPCX-talin WT	Forward: GCC CAT AAA GCT TAT ACG AAT TCA CCA TGG TTG CGC TTT CGC<br>Reverse: GAG ACT AAA TAA AAT CTT TTA TTT TAT CGA TTT AG
pLPCX-talin head	Forward: GCC CAT AAA GCT TAT ACG AAT TCA CCA TGG TTG CGC TTT CGC<br>Reverse: CGC CTT TGC TCA CTC CTG CGG CCG CTC CCA TGT GTC CTC GGT GCA TCT GG
pLPCX-talin DDdel	Forward: GGA AAT AAA AGC AGC AGC TC<br>Reverse: CGC CTT TGC TCA CTC CTG CGG CCG CTC CGT CTT CGA AGG CTG CAG CTT TCT G
pLPCX-talin R2526G	Forward1: GGA AAT AAA AGC AGC AGC TC<br>Reverse1: CTG CTG CCC GAT CTG GGC GAG CTT TTT CC

	Forward2: CCC AGA TCG GGC AGC AGC AGT ACA AGT TCT TG<br>Reverse2: GAG ACT AAA TAA AAT CTT TTA TTT TAT CGA TTT AG
pLPCX-talin M3	Forward: GCC CAT AAA GCT TAT ACG AAT TCA CCA TGG TTG CGC TTT CGC<br>Reverse: CGC CTT TGC TCA CTC CTG CGG CCG CTC CGT GCT CGT CTC GA
pLPCX-talin R2mut (A680E, K687E)	Forward1: GCC CAT AAA GCT TAT ACG AAT TCA CCA TGG TTG CGC TTT CGC<br>Reverse1: GCC TTG AGG ACC AGG GCC TCT GCA GCA CTT G<br>Forward2: CCC TGG TCC TCA AGG CCG AGA GTG TGG CCC<br>Reverse2: GGG CTT AAG CTT GCC ATC TCG AGC TG
pLPCX-talin R11mut (A2126E, K2133E)	Forward1: GGA AAT AAA AGC AGC AGC TC<br>Reverse1: CCA CTG TCT TGA GCA ATG ACT CCA CAT TGG TCA<br>Forward2: TCA TTG CTC AAG ACA GTG GAG GCT GTG GAA GA<br>Reverse2: CGC CTT TGC TCA CTC CTG CGG CCG CTC CGT GCT CGT CTC GA
pLPCX-talin R2R11mut	Forward1: GCC CAT AAA GCT TAT ACG AAT TCA CCA TGG TTG CGC TTT CGC<br>Reverse1: GCC TTG AGG ACC AGG GCC TCT GCA GCA CTT G<br>Forward2: CCC TGG TCC TCA AGG CCG AGA GTG TGG CCC<br>Reverse2: GGG CTT AAG CTT GCC ATC TCG AGC TG<br>Forward3: GGA AAT AAA AGC AGC AGC TC<br>Reverse3: CCA CTG TCT TGA GCA ATG ACT CCA CAT TGG TCA<br>Forward4: TCA TTG CTC AAG ACA GTG GAG GCT GTG GAA GA<br>Reverse4: CGC CTT TGC TCA CTC CTG CGG CCG CTC CGT GCT CGT CTC GA
pLPCX-K2 WT	Forward: GCC CAT AAA GCT TAT ACG AAT TCA CCA TGG TGA GCA AGG GCG AGG AGG<br>Reverse: GAG ACT AAA TAA AAT CTT TTA TTT TAT CGA TTC ACA CCC AAC CAC TGG TAA GTT TG
pLPCX-K2 GLKE	Forward: GCC CAT AAA GCT TAT ACG AAT TCA CCA TGG TGA GCA AGG GCG AGG AGG<br>Reverse: GAG ACT AAA TAA AAT CTT TTA TTT TAT CGA TTC ACA CCC AAC CAC TGG TAA GTT TG
pLPCX-mCherry	Forward: GCC CAT AAA GCT TAT ACG AAT TCA CCA TGG TGA GCA AGG GCG AGG AGG<br>Reverse: GAG ACT AAA TAA AAT CTT TTA TTT TAT CGA TTC ACT TGT ACA GCT CGT CCA TGC CGC CG

Restriction sites are underlined.

References

1 Dedden, D. *et al.* The Architecture of Talin1 Reveals an Autoinhibition Mechanism. *Cell* **179**, 120-131 e113, doi:10.1016/j.cell.2019.08.034 (2019).

2 Moore, D. T. *et al.* Affinity of talin-1 for the beta3-integrin cytosolic domain is modulated by its phospholipid bilayer environment. *Proc Natl Acad Sci U S A* **109**, 793-798, doi:10.1073/pnas.1117220108 (2012).

3 Fischer, L. S. *et al.* Quantitative single-protein imaging reveals molecular complex formation of integrin, talin, and kindlin during cell adhesion. *Nat Commun* **12**, 919, doi:10.1038/s41467-021-21142-2 (2021).

Uncropped Images

Supplementary Figure 1a

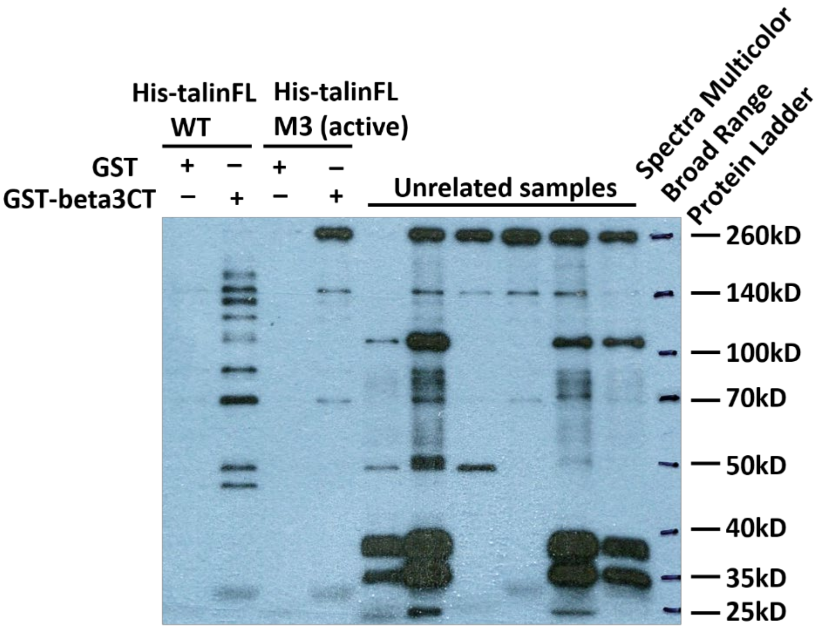

Supplementary Figure 1c

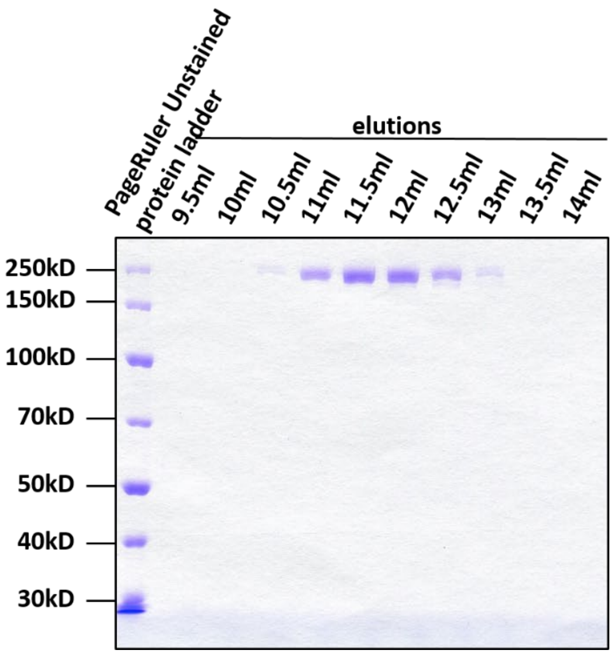

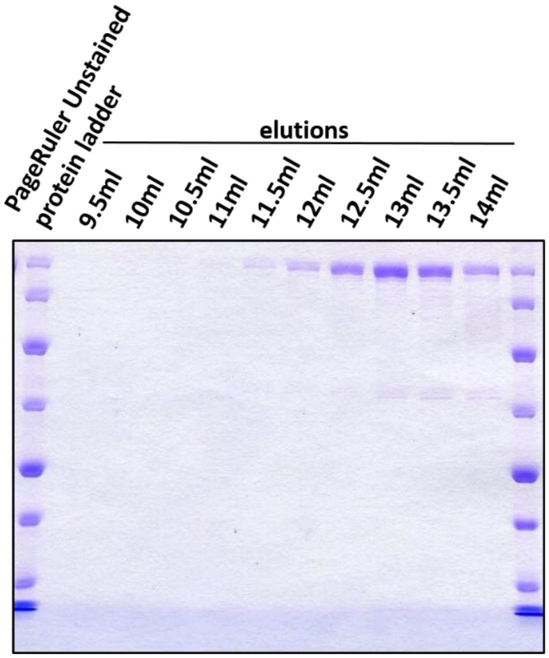

Supplementary Figure 2c

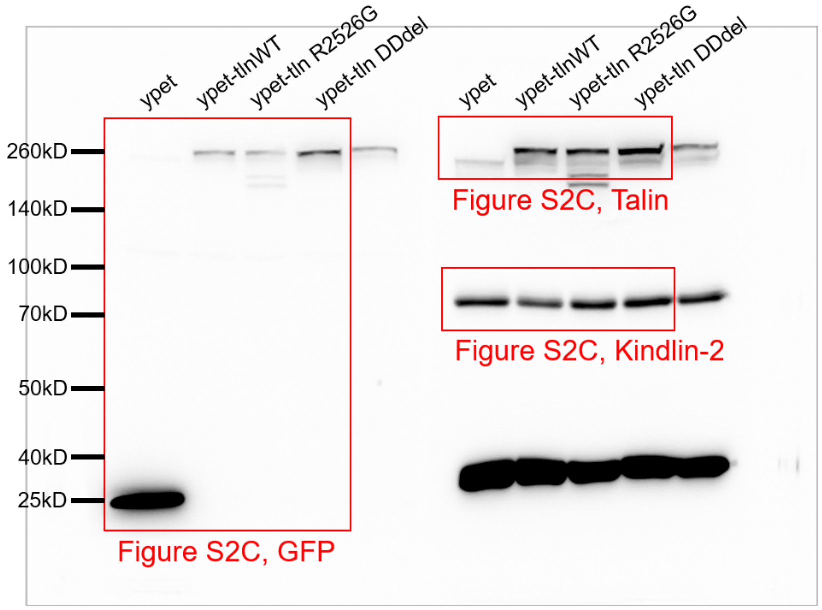

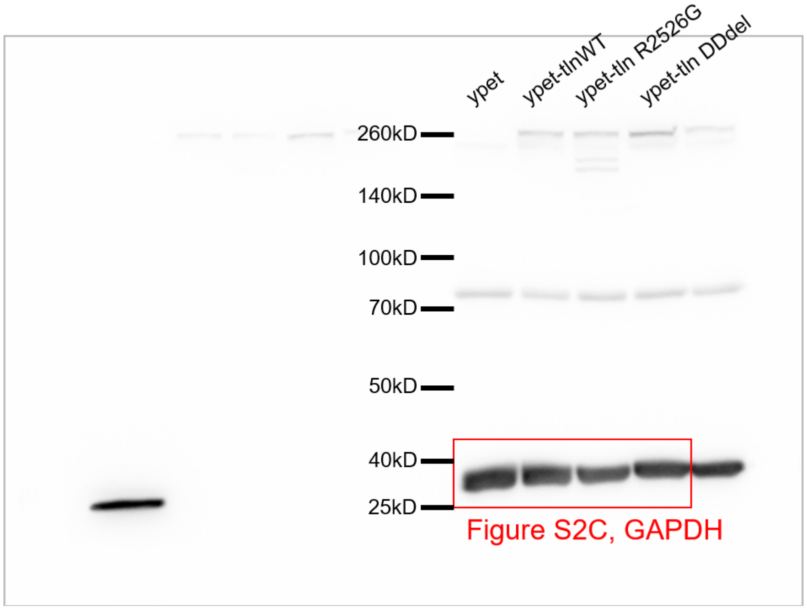

Supplementary Figure 3b

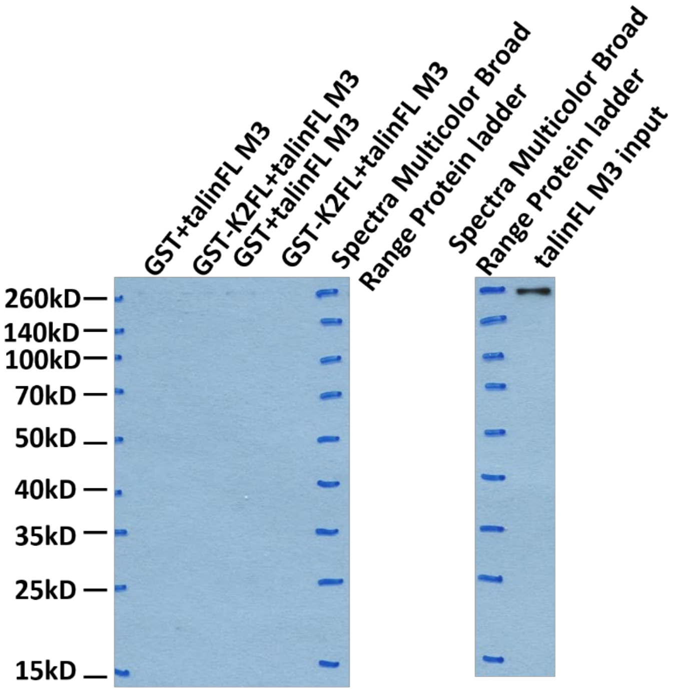

Supplementary Figure 3f

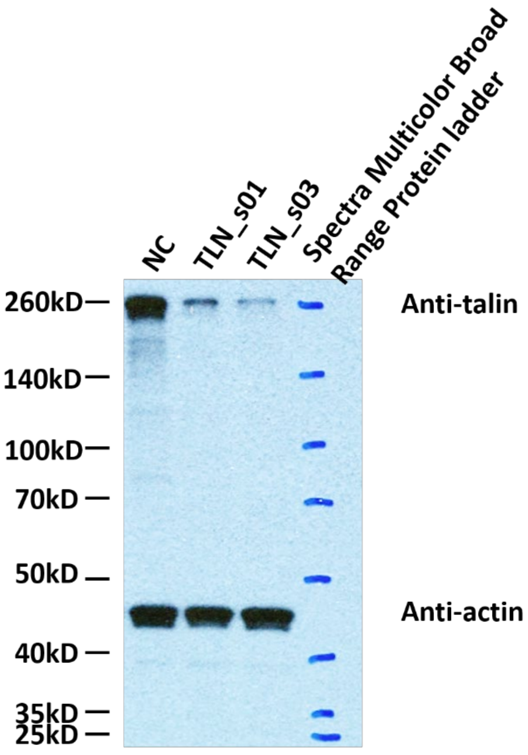

Supplementary Figure 3i

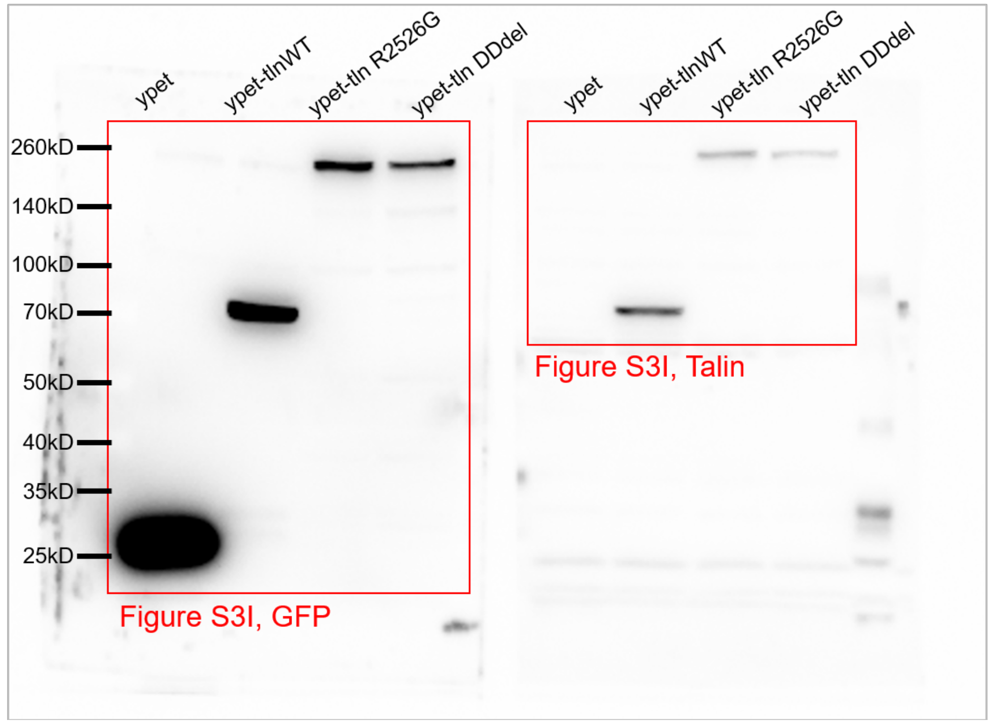

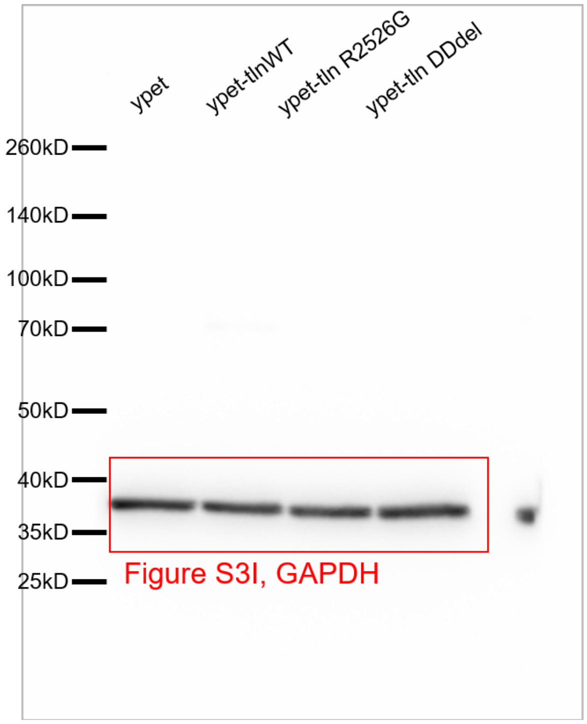

Supplementary Figure 4a

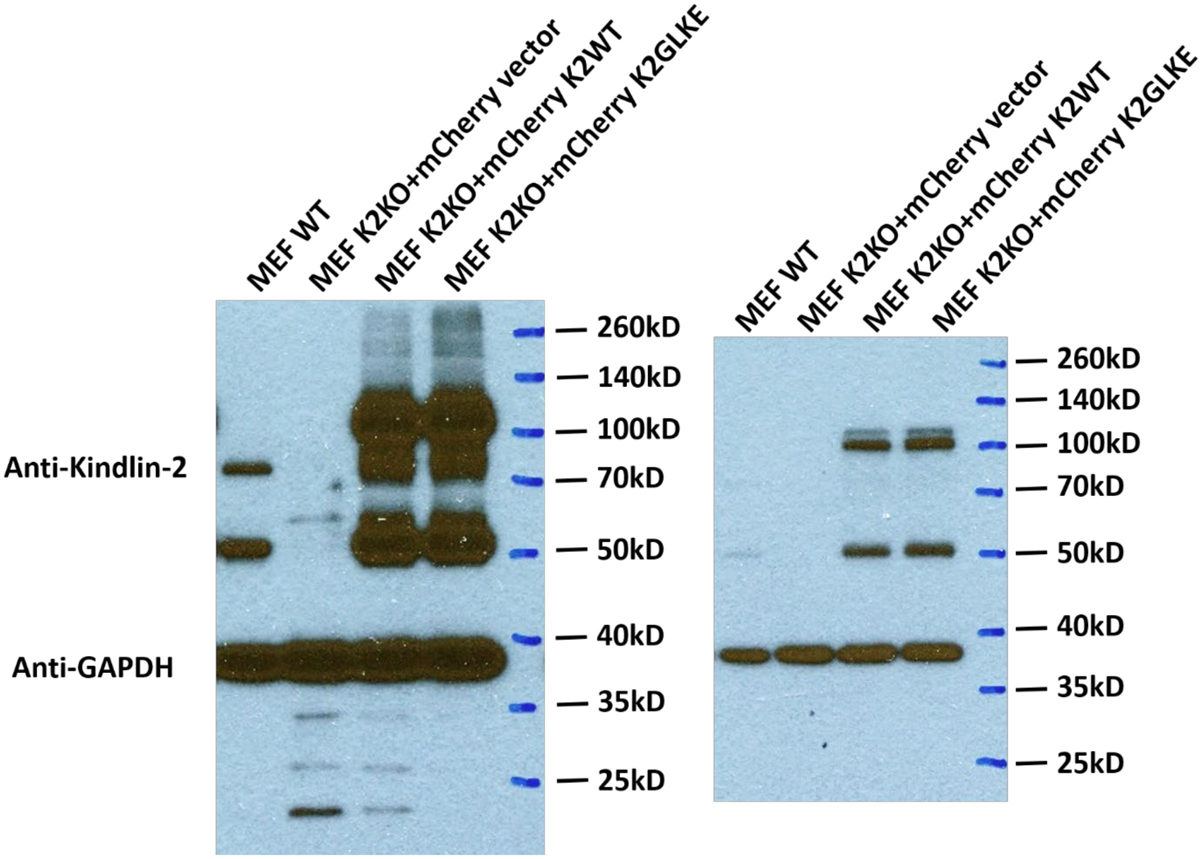

Supplementary Figure 4c

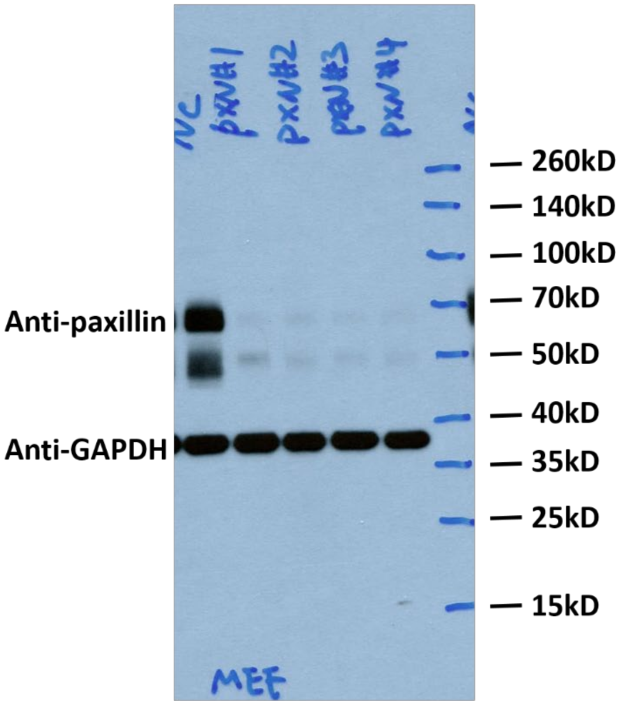

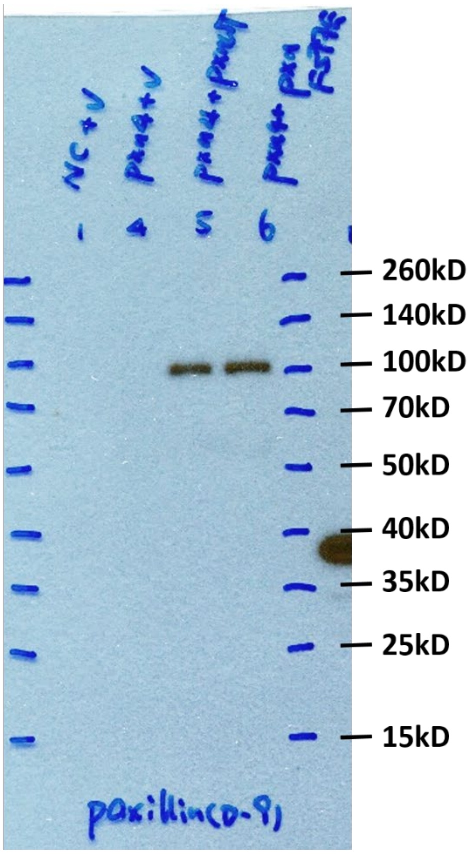

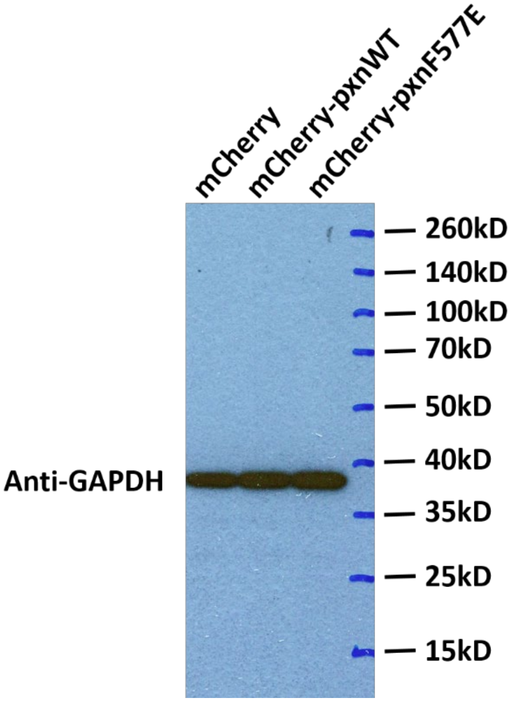

Supplementary Figure 5d

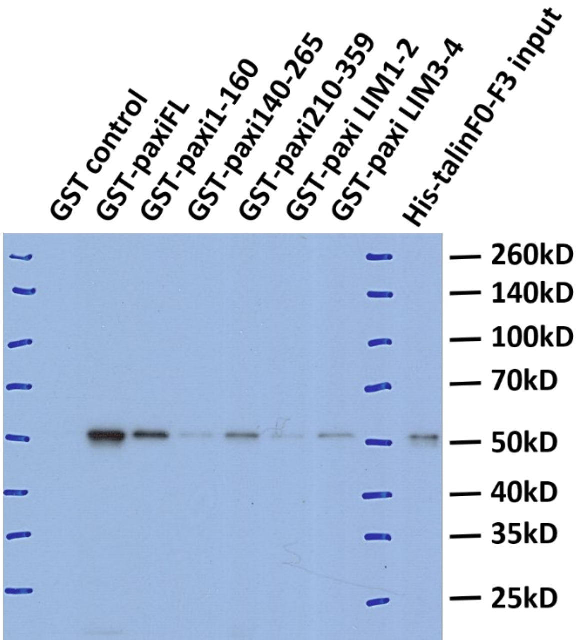

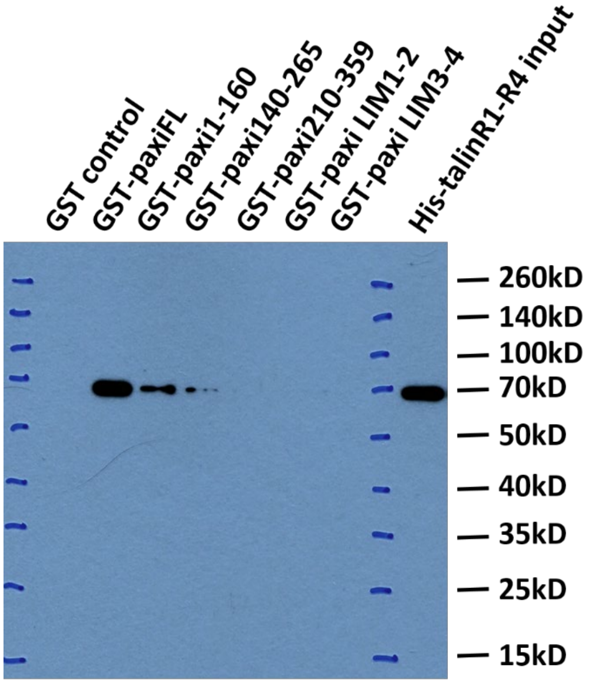

Supplementary Figure 12a

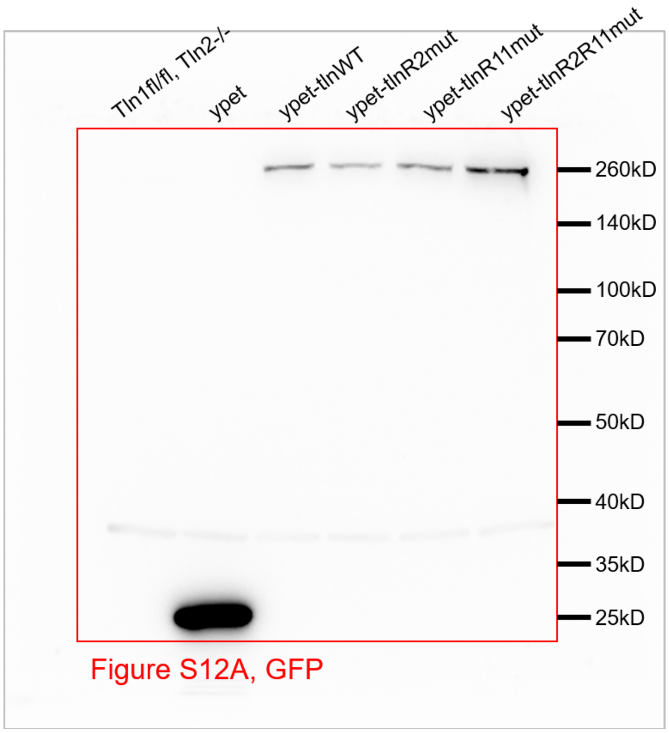

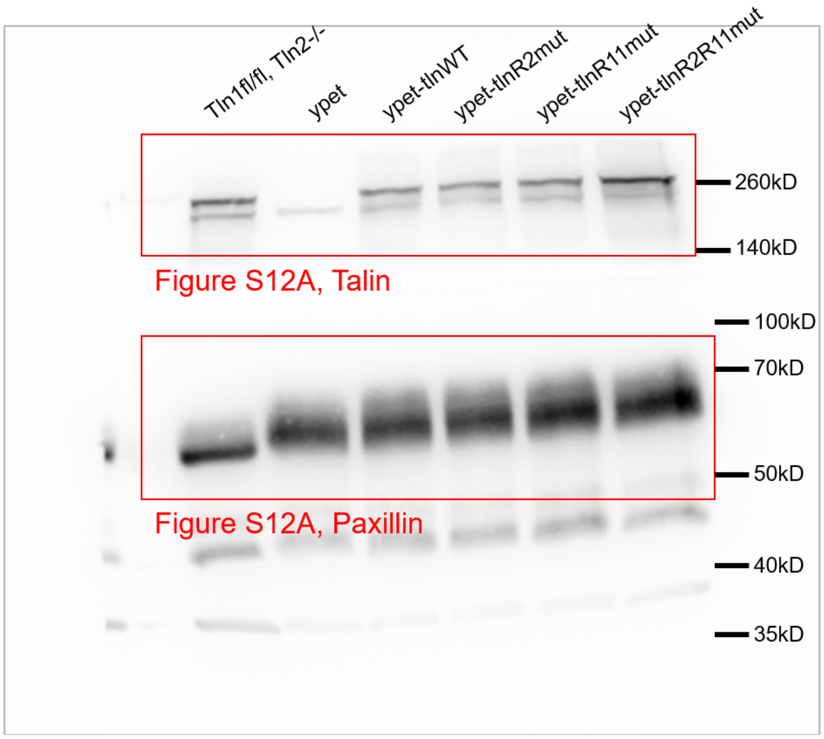

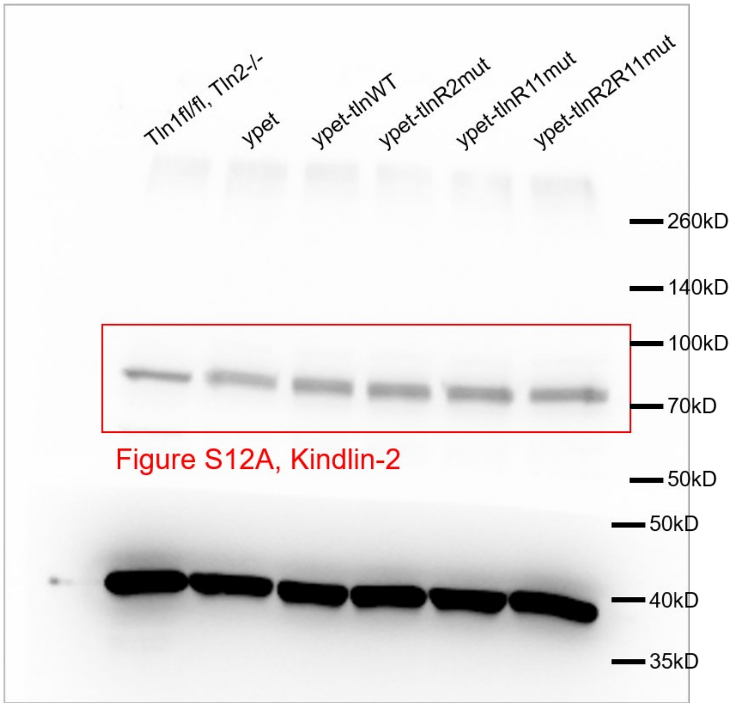
Tln1fl/fl, Tln2-/-
ypet
ypet-tlnWT
ypet-tlnR2mut
ypet-tlnR11mut
ypet-tlnR2R11mut
260kD
140kD
100kD
70kD
50kD
50kD
40kD
35kD
Figure S12A, Kindlin-2

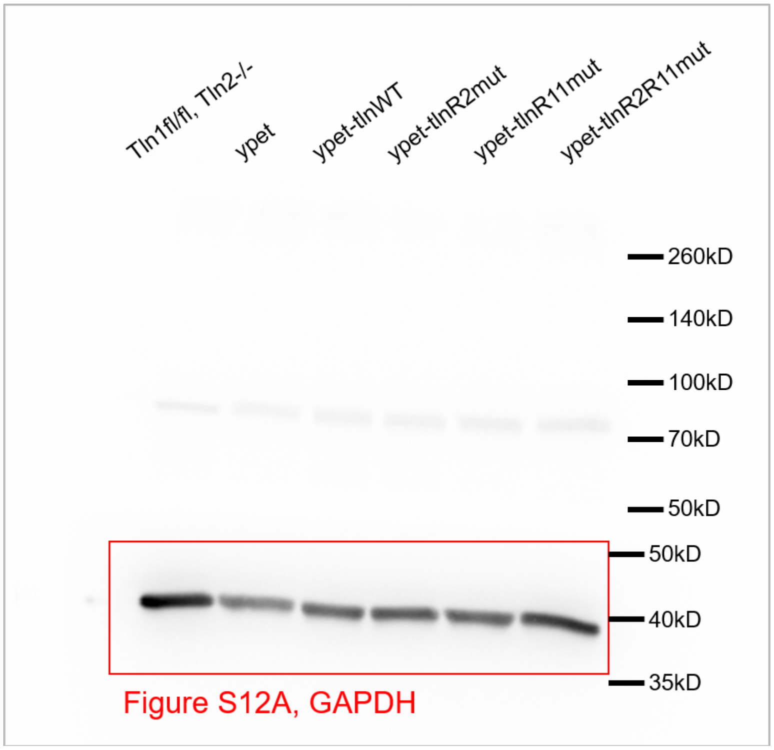
Tln1fl/fl, Tln2-/-
ypet
ypet-tlnWT
ypet-tlnR2mut
ypet-tlnR11mut
ypet-tlnR2R11mut
260kD
140kD
100kD
70kD
50kD
50kD
40kD
35kD
Figure S12A, GAPDH
